# Supplementary material for: Tpr Misregulation in Hippocampal Neural Stem Cells in Mouse Models of Alzheimer’s Disease
Source: Cells. 2023 Dec 1;12(23):2757. doi: 10.3390/cells12232757 (PMC10706632; doi:10.3390/cells12232757)
Supplement: Supplementary file 1 [file cells-12-02757-s001.zip › cells-2633159-supplementary.pdf]

cells

MDPI

Article

Tpr Misregulation in Hippocampal Neural Stem Cells in Mouse Models of Alzheimer's Disease

Subash C. Malik [1,2], Jia-Di Lin [1,2], Stephanie Ziegler-Waldkirch [3], Stefan Tholen [4], Sachin S. Deshpande [1,2], Marius Schwabenland [5], Oliver Schilling [4], Andreas Vlachos [6,7,8], Melanie Meyer-Luehmann [3,8] and Christian Schachtrup [1,8,*]

[1] Institute of Anatomy and Cell Biology, University of Freiburg, 79104 Freiburg, Germany; subash.chandra.malik@ki.se (S.C.M.); jia-di.lin@anat.uni-freiburg.de (J.-D.L.); deshpande03@gmail.com (S.S.D.)
[2] Faculty of Biology, University of Freiburg, 79104 Freiburg, Germany
[3] Department of Neurology, Medical Center, Faculty of Medicine, University of Freiburg, 79106 Freiburg, Germany; stephanie.waldkirch@uniklinik-freiburg.de (S.Z.-W.); melanie.meyer-luehmann@uniklinik-freiburg.de (M.M.-L.)
[4] Institute of Surgical Pathology, Medical Center, University of Freiburg, 79106 Freiburg, Germany; stefan.tholen@uniklinik-freiburg.de (S.T.); oliver.schilling@uniklinik-freiburg.de (O.S.)
[5] Institute of Neuropathology, University of Freiburg, 79106 Freiburg, Germany
[6] Department of Neuroanatomy, Institute of Anatomy and Cell Biology, Faculty of Medicine, University of Freiburg, 79104 Freiburg, Germany; andreas.vlachos@anat.uni-freiburg.de
[7] Center BrainLinks-BrainTools, University of Freiburg, 79110 Freiburg, Germany
[8] Center for Basics in Neuromodulation (NeuroModul Basics), Faculty of Medicine, University of Freiburg, 79106 Freiburg, Germany
* Correspondence: christian.schachtrup@anat.uni-freiburg.de

Supplemental Figures and Tables

Table of Contents

S1. **Supplemental Figures**

S1.1 Tpr phosphorylation determines the subcellular localization in NSPCs of the hippocampus

S1.2 Increased Tpr expression precedes hippocampal neurogenesis in 5xFAD mice

S1.3 Tpr and P-Tpr interactome in NSPCs

S1.4 Tpr siRNA knockdown in primary NSPCs

S2. **Supplemental Tables**

S2.1 Supplemental Table S1: Tpr interacting proteins of WT adult NSPCs.

S2.2 Supplemental Table S2: P-Tpr interacting proteins of WT adult NSPCs.

S1. Supplemental Figures

S1.1 Tpr phosphorylation determines the subcellular localization in NSPCs of the hippocampus

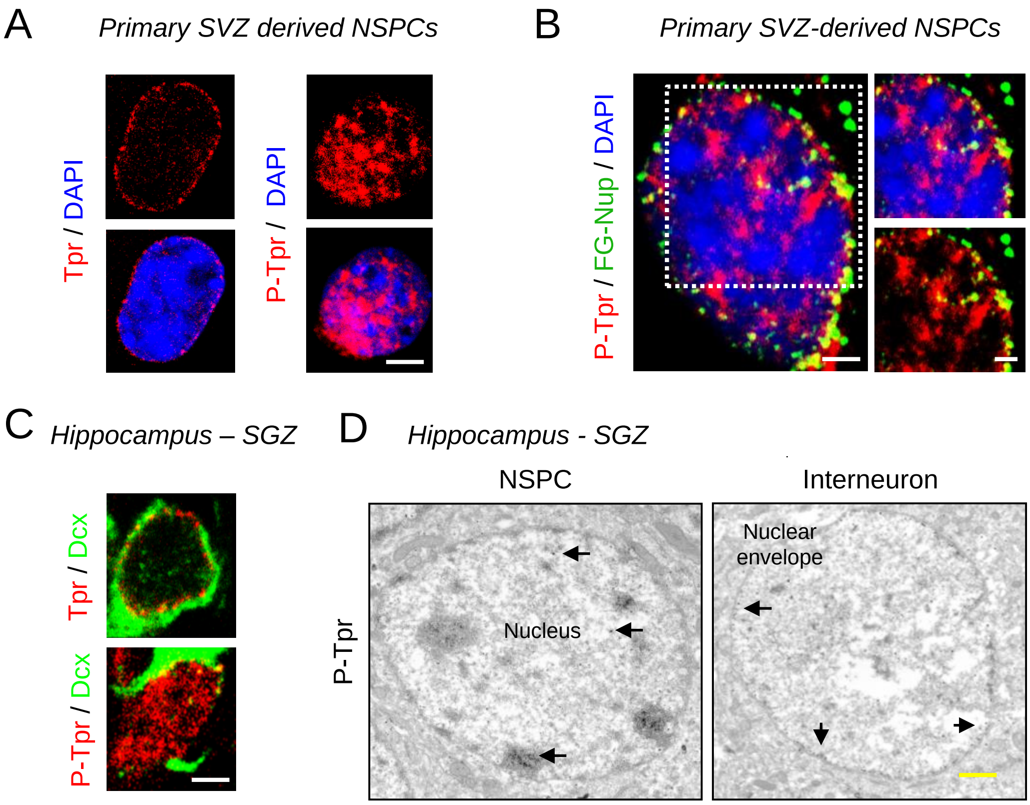

Supplemental Figure S1. Tpr phosphorylation determines the subcellular localization in NSPCs of the hippocampus.

(A) Immunolabeling for P-Tpr (red, right) and Tpr (red, left) in NSPCs *in vitro*. Nuclei are stained with DAPI (blue). Representative image from three independent experiments (A total number of 20 cells per Tpr or P-Tpr was analyzed). Scale bar: 3.3 µm. **(B)** Immunolabeling for P-Tpr (red) and FG-Nup (green) in NSPCs *in vitro*. Enlargements at the right indicate nuclear P-Tpr (red) localization in NSPCs. Nuclei are stained with DAPI (blue). Scale bars: 2 µm. **(C)** Immunolabeling for P-Tpr (red, right) or Tpr (red, left) in combination with DCX (green, marker for neuroblasts) in hippocampal SGZ NSPCs in adult WT mice. Representative image from three independent experiments (A total number of 20 cells per Tpr+DCX+ or P-Tpr+DCX+ cells was analyzed). Scale bar: 3.3 µm. **(D)** Electron microscopy determining the P-Tpr localization in NSPCs of the hippocampal SGZ in comparison to hippocampal interneurons in adult WT mice. Black arrows indicating nuclear P-Tpr (left) and nuclear envelope P-Tpr (right). Representative image from three independent experiments (A total number of 10 cells per NSPCs or interneurons for P-Tpr localization was analyzed). Scale bar: 1 µm.

S1.2 Increased Tpr expression precedes hippocampal neurogenesis in 5xFAD mice

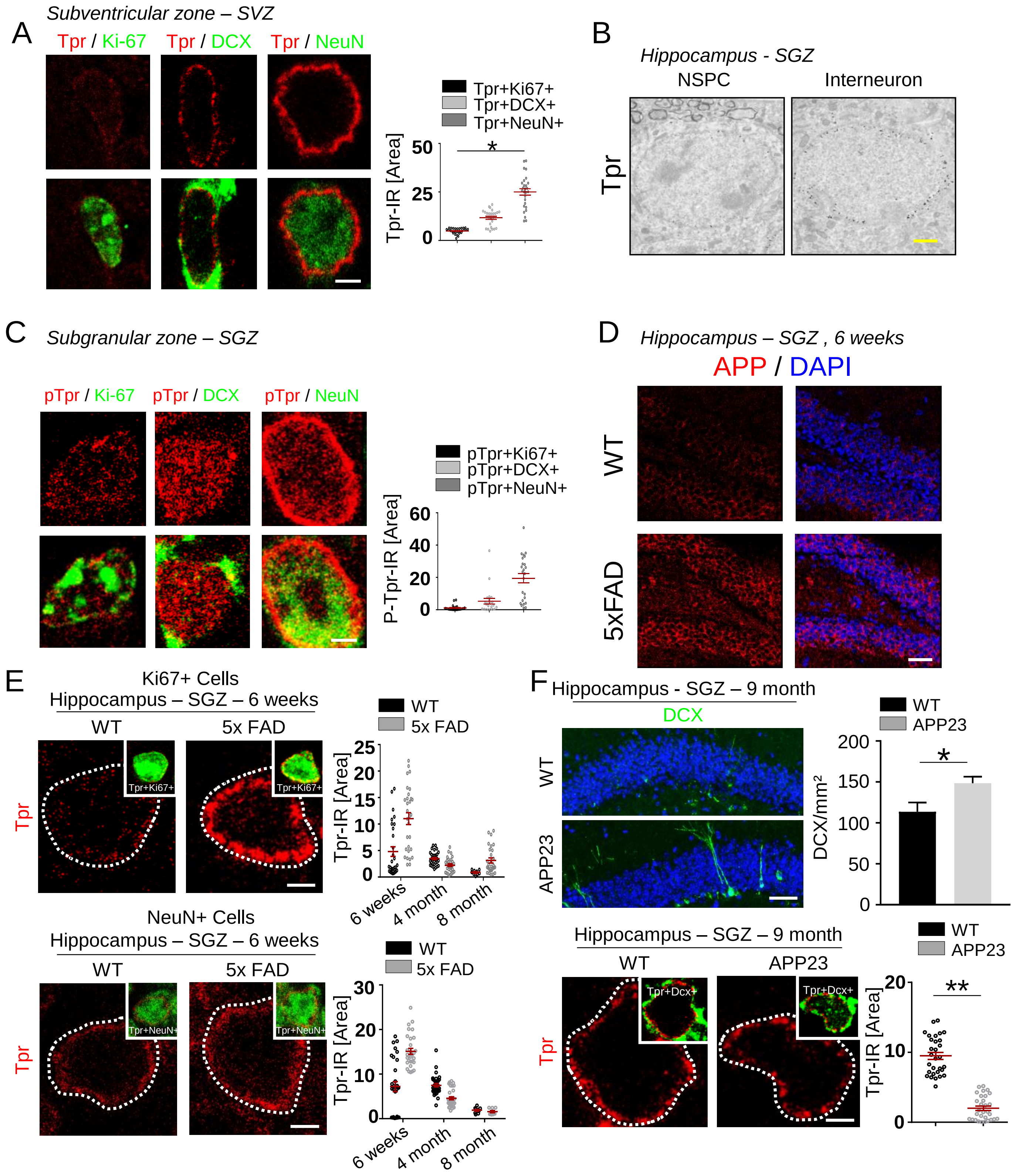

Supplemental Figure S2. Increased Tpr expression precedes hippocampal neurogenesis in 5xFAD mice.

(A) Immunolabeling for Tpr (red) and Ki-67, DCX, and NeuN (all red) in individual cells in the SVZ of adult WT mice. Quantification of Tpr immunoreactivity in Ki-67+, DCX+ and NeuN+ cells in the SVZ of adult WT mice (n = 3 mice, a total number of 27 single cells per Tpr+Ki-67+, Tpr+DCX+ or Tpr+NeuN+ condition were analyzed). Scale bar: 2 μm. Values are mean ± SEM (P-values calculated by one-way ANOVA and Bonferroni's multiple comparisons test, $^*P < 0.05$). **(B)** Electron microscopy determining the Tpr expression in NSPCs of the hippocampal SGZ in comparison to hippocampal interneurons in adult WT mice (n = 2 mice, a total number of 10 single cells per NSPCs or interneurons for Tpr localization were analyzed). Scale bar: 1 μm. **(C)** Immunolabeling for P-Tpr (red) and Ki-67, DCX, and NeuN (all green) in individual cells in the SGZ of adult WT mice. Quantification of P-Tpr immunoreactivity in Ki-67+, DCX+ and NeuN+ cells in the SGZ of adult WT mice (n = 4 mice, a total number of 22-24 single cells per P-Tpr+Ki-67+, P-Tpr+DCX+ or P-Tpr+NeuN+ condition were analyzed). Scale bar: 2 μm. Values are mean ± SEM. **(D)** Immunolabeling for APP (red) in the hippocampus of 5xFAD mice in comparison to WT mice at 6 weeks of age. Representative images from n=3 mice. Scale bar: 50 μm. **(E)** Immunolabeling for Tpr (red) in individual cells in the SGZ of the hippocampus of 5xFAD mice compared to control mice sacrificed at 6 weeks of age (Inlets: Tpr+Ki67+ cells (top) and Tpr+NeuN+ cells (bottom) in the SGZ of the hippocampus). Scale bars: 2 μm. Quantification of Tpr immunoreactivity in Ki67+ cells and NeuN+ cells, respectively, in the hippocampal SGZ of 5xFAD mice compared to control mice at 6 weeks and 4 and 8 months of age (n =3 mice, a total number of 30-32 single cells of Ki67+ or NeuN+ cells at 6 weeks and 4 month in WT and 5X FAD mice were analyzed and a total number of 9 (WT mice) and 29 (5X FAD mice) single cells of Ki67+ or NeuN+ cells were analyzed at 8 month of age. **(F)** Immunolabeling for DCX (green) in the hippocampus of APP23 mice compared to control mice sacrificed at 9 months of age (top). Scale bar: 50 μm. Quantification of DCX+ cells in the hippocampal SGZ of APP23 mice compared to control mice at 9 months of age (n = 3 mice). Values are mean ± SEM (P-values calculated by unpaired Student's *t* test, $^*P < 0.05$. Immunolabeling for Tpr (red) in the SGZ of the hippocampus of APP23 mice compared to control mice sacrificed at 9 months of age (Inlets: only Tpr+DCX+ cells in the SGZ of the hippocampus were analyzed) (n = 3 mice, a total number of 30 single cells for Tpr immunoreactivity in the hippocampal SGZ in WT and APP23 mice were analyzed). Scale bar: 2 μm. Quantification of Tpr immunoreactivity in DCX+ cells in the hippocampal SGZ of APP23 mice compared to control mice at 9 months of age. Values are mean ± SEM (P-values calculated by unpaired Student's *t* test, $^{**}P < 0.01$).

S1.3 Tpr and P-Tpr interactome in NSPCs

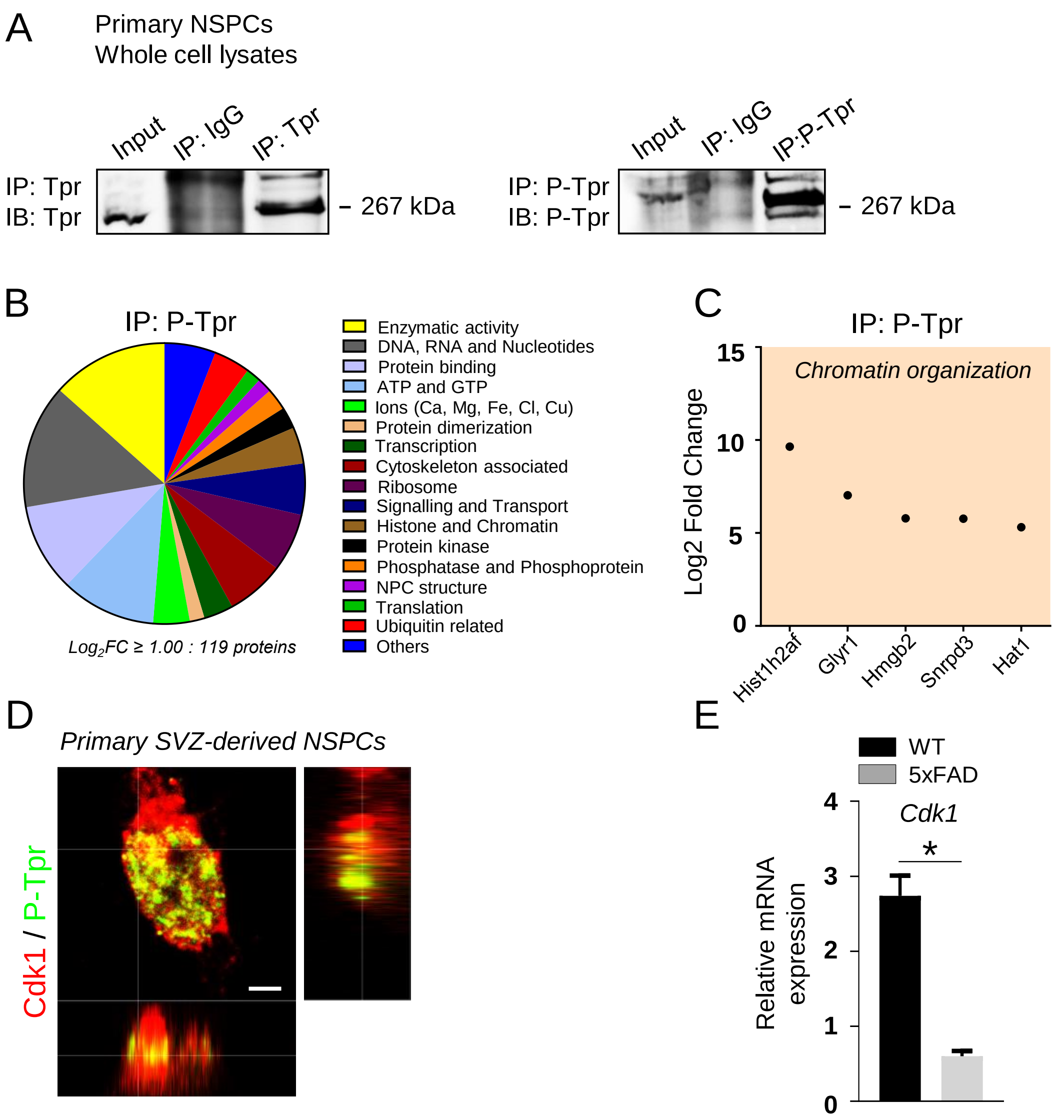

Supplemental Figure S3. Tpr and P-Tpr interactome in NSPCs.

(A) Co-immunoprecipitation (Co-IP) with Tpr and P-Tpr in whole-cell lysates of SVZ-derived NSPCs. Representative immunoblots from three independent experiments. **(B)** Pie chart of identified P-Tpr interaction partner in NSPCs classified via the Gene Ontology (GO) nomenclature. **(C)** Plot showing top five interaction partners with a log2 fold value ≥ 5.0 for P-Tpr involved in chromatin organization and control of stem cell fate. **(D)** Immunolabeling for Cdk1 (red) in combination with P-Tpr (red) revealing colocalization of P-Tpr with Cdk1 in the nucleus (yellow). Representative image from two independent experiments (a total number of 10 cells was analyzed). Scale bar: 3 µm. (E) Decreased Cdk1 gene expression in hippocampal SGZ tissue of 8-month-old 5xFAD mice compared to control mice ($n = 2$ mice per genotype) performed by RT-PCR analysis performed in duplicate (P-values calculated by student's t test, *$P < 0.05$).

S1.4 Tpr siRNA knockdown in primary NSPCs

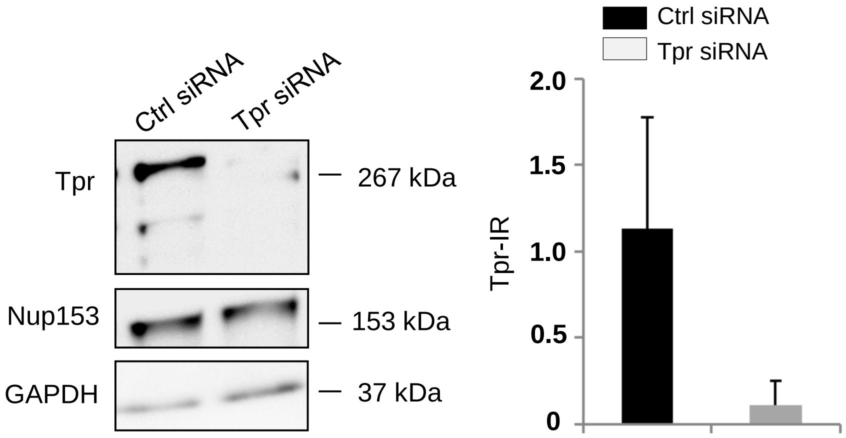

Supplemental Figure S4. Tpr siRNA knockdown in primary NSPCs.

Protein expression of Tpr using lysate of NSPCs 1 day after electroporation with Tpr siRNA and scramble siRNA (control) determined by western blotting. Quantification of Tpr immunoreactivity in Tpr-depleted NSPCs compared to control cells. Ctrl, control; siRNA, small interfering RNA. Representative immunoblots are shown from two independent experiments.

S2. Supplemental Tables

S*2.1 Supplemental Table S1: Tpr-interacting proteins of WT adult NSPCs.*
Supplemental Table S1: Table showing 759 Tpr interacting proteins of WT adult NSPCs with a Log2 FC > 1.0.

**Protein IDs**	**Protein names**	**Gene names**	**Unique peptides**	**Sequence coverage [%]**	**Log2 fold-change (FC) of LFQ intensity (a-TPR/IgG)**
**P25976**	Nucleolar transcription factor 1	Ubtf	53	48,8	1,569809859
**Q7JJ13**	Bromodomain-containing protein 2	Brd2	44	54,6	1,830780269
**Q3U0V1**	Far upstream element-binding protein 2	Khsrp	40	57	1,197171454
**E9Q5C9**		Nolc1	44	43,7	1,329159023
**Q8BTI8**	Serine/arginine repetitive matrix protein 2	Srrm2	41	21,3	1,634042181
**Q9JIX8**	Apoptotic chromatin condensation inducer in the nucleus	Acin1	39	33,5	1,229755172
**Q91VN6**	Probable ATP-dependent RNA helicase DDX41	Ddx41	38	62,9	1,441561598
**Q8BHL3**	TBC1 domain family member 10B	Tbc1d10b	37	47	1,665057358
**O54774**	AP-3 complex subunit delta-1	Ap3d1	33	34,7	1,577511208
**Q8K2F0;**	Bromodomain-containing protein 3	Brd3	27	40,8	1,996823053

**Q91Y44**					
**A2AQ19**	RNA polymerase-associated protein RTF1 homolog	Rtf1	28	38	2,320407779
**P62908**	40S Ribosomal protein S3	Rps3	26	83,5	1,122783141
**Q5F2E8**	Serine/threonine-protein kinase TAO1	Taok1	22	25	2,043417601
**A0A1Y7VKY1; F6YVP7; P62270**	40S Ribosomal protein S18	Gm10260; Rps18	23	62,5	1,230576431
**Q6NSQ7**	Protein LTV1 homolog	Ltv1	22	54	2,934466665
**P97868**	E3 ubiquitin-protein ligase RBBP6	Rbbp6	22	19,4	2,121588475
**O08784**	Treacle protein	Tcof1	22	19,1	1,714693876
**Q3UMU9**	Hepatoma-derived growth factor-related protein 2	Hdgfrp2	22	37,1	1,355634343
**Q3V1V3**	ESF1 homolog	Esf1	21	25,7	2,582366338
**Q8VDM6**	Heterogeneous nuclear ribonucleoprotein U-like protein 1	Hnrnpul1	20	33,8	1,330628683
**P23116**	Eukaryotic translation initiation factor 3 subunit A	Eif3a	19	17,6	1,67169448
**Q8R1B4**	Eukaryotic translation initiation factor 3 subunit C	Eif3c	19	22,1	1,156168458
**P55096**	ATP-binding cassette sub-family D member 3	Abcd3	19	32,5	1,143131998
**Q8BGS1**	Band 4.1-like protein 5	Epb41l5	18	36,1	1,683164414
**Q9QZF2**	Glypican-1; Secreted glypican-1	Gpc1	17	39,3	1,109327499
**Q9D6Z1**	Nucleolar protein 56	Nop56	17	32,2	1,075016063
**P30415**	NK-tumor recognition protein; Putative peptidyl-prolyl cis-trans isomerase	Nktr	16	16,4	1,904342155
**P63089**	Pleiotrophin	Ptn	16	61,9	1,6521214
**P62852**	40S Ribosomal protein S25	Rps25	16	55,2	1,211770512
**Q8C5N3; A2AK44; A2AK42**	Pre-mRNA-splicing factor CWC22 homolog	Cwc22; Gm13695; Gm13697	15	18,1	2,557003563
**Q52KI8**	Serine/arginine repetitive matrix protein 1	Srrm1	15	17,1	2,300792903
**P70318**	Nucleolysin TIAR	Tial1	10	46,9	1,764555209
**Q9DC71**	28S Ribosomal protein S15, mitochondrial	Mrps15	15	45,7	1,48289261
**Q91WJ8**	Far-upstream element-binding protein 1	Fubp1	15	32,4	1,426133106

**O70503**	Very-long-chain 3-oxoacyl-CoA reductase	Hsd17b12	15	49	1,245107691
**Q64512**	Tyrosine-protein phosphatase non-receptor type 13	Ptpn13	14	7,7	4,684448381
**P40201; E9PZM4**	Chromodomain-helicase-DNA-binding protein 1	Chd1	14	11,7	2,623603558
**P97376**	Protein FRG1	Frg1	14	41,1	2,109152979
**Q60932**	Voltage-dependent anion-selective channel protein 1	Vdac1	14	63,5	1,85475873
**Q7TNV0**	Protein DEK	Dek	14	28,7	1,749429026
**P11031**	Activated RNA polymerase II transcriptional coactivator p15	Sub1	14	63	1,487430892
**Q6ZQ38**	Cullin-associated NEDD8-dissociated protein 1	Cand1	13	13,4	1,451981026
**Q9WVA3**	Mitotic checkpoint protein BUB3	Bub3	14	51,2	1,271692235
**Q6P542**	ATP-binding cassette sub-family F member 1	Abcf1	13	22,6	3,805878161
**D3Z0M9**		Ddx23	13	20	1,667425576
**A2AR02**	Peptidyl-prolyl cis-trans isomerase G	Ppig	13	15,2	1,40428315
**Q8BKC5**	Importin-5	Ipo5	13	16,3	1,394805135
**Q9ERI5**	Bifunctional arginine demethylase and lysyl-hydroxylase JMJD6	Jmjd6	13	42,2	1,23916841
**Q80WJ7**	Protein LYRIC	Mtdh	13	35,2	1,020763496
**Q3UFY8**	Mitochondrial ribonuclease P protein 1	Trmt10c	12	36	4,055607206
**Q8K0C4**	Lanosterol 14-alpha demethylase	Cyp51a1	12	26,6	3,187238185
**Q8BMC4**	Nucleolar protein 9	Nop9	12	28,1	1,953765849
**O55143; Q8R429; Q64518**	Sarcoplasmic/endoplasmic reticulum calcium ATPase 2	Atp2a2	12	17,4	1,93219986
**Q9ESX5**	H/ACA ribonucleoprotein complex subunit 4	Dkc1	12	33,8	1,570576343
**Q9CR62**	Mitochondrial 2-oxoglutarate/malate carrier protein	Slc25a11	12	40,1	1,544761529
**Q9R0N3**	Synaptotagmin-11	Syt11	12	36,7	1,106520452
**Q9DBG6**	Dolichyl-diphosphooligosaccharide--protein glycosyltransferase subunit 2	Rpn2	12	31,1	1,075666201
**A2A6A1**	G patch domain-containing protein 8	Gpatch8	11	8,8	5,127961262
**Q9JKF1**	Ras GTPase-activating-like protein IQGAP1	Iqgap1	11	10,3	4,714841555

**Q99LX5**	Multiple myeloma tumor-associated protein 2 homolog	Mmtag2	11	47,7	3,623905227
**Q9Z1G4**	V-type proton ATPase 116 kDa subunit a isoform 1	Atp6v0a1	11	16,6	2,616872227
**Q9R020**	Zinc-finger Ran-binding domain-containing protein 2	Zranb2	11	31,5	2,378296745
**P67984**	60S Ribosomal protein L22	Rpl22	10	70,3	1,606827152
**Q07646**	Mesoderm-specific transcript protein	Mest	11	36,7	1,565936918
**Q8VD75**	Huntingtin-interacting protein 1	Hip1	11	14,2	1,520339636
**Q9D4J7**	PHD finger protein 6	Phf6	11	39,3	1,304397979
**P51655**	Glypican-4; Secreted glypican-4	Gpc4	10	26	1,208546295
**P52019**	Squalene monooxygenase	Sqle	11	28,7	1,050630992
**Q9WVK4**	EH domain-containing protein 1	Ehd1	9	29,2	12,68575623
**Q64133**	Amine oxidase [flavin-containing] A	Maoa	10	25,7	2,520346966
**Q9CY27**	Very-long-chain enoyl-CoA reductase	Tecr	10	26,3	1,814151222
**Q08288**	Cell growth-regulating nucleolar protein	Lyar	10	24,2	1,78693155
**E9Q557**	Desmoplakin	Dsp	10	4,2	1,514336417
**Q60930**	Voltage-dependent anion-selective channel protein 2	Vdac2	10	43,7	1,283168863
**Q99LC3**	NADH dehydrogenase [ubiquinone] 1 alpha subcomplex subunit 10, mitochondrial	Ndufa10	9	31	13,34789814
**Q91UZ1**	Phosphoinositide phospholipase C	Plcb4	9	8,5	11,39156582
**P62141**	Serine/threonine-protein phosphatase PP1-beta catalytic subunit	Ppp1cb	2	38,5	10,60917874
**Q99P88**	Nuclear pore complex protein Nup155	Nup155	9	9,3	4,256093489
**Q8K224**	N-acetyltransferase 10	Nat10	9	10,1	3,653141754
**Q6NV83**	U2 snRNP-associated SURP motif-containing protein	U2surp	9	10,3	3,405930634
**Q8QZY1**	Eukaryotic translation initiation factor 3 subunit L	Eif3l	9	20,4	2,826637372
**Q3UQU0**	Bromodomain-containing protein 9	Brd9	9	21,5	2,730081234
**Q9DBR0**	A-Kinase anchor protein 8	Akap8	9	23,3	2,695930757
**Q5F2E7**	Nuclear fragile X mental retardation-interacting protein 2	Nufip2	9	21,4	2,665465576
**Q9CS00**	Cactin	Cactin	9	15,2	2,494941284
**P06151;**	L-Lactate dehydrogenase A chain	Ldha	9	29,5	2,221730093

**P00342**					
**Q3TVI8**	Pre-B-cell leukemia transcription factor-interacting protein 1	Pbxip1	9	16	2,097903481
**Q05D44**	Eukaryotic translation initiation factor 5B	Eif5b	9	11,3	1,749933916
**Q9Z1T1**	AP-3 complex subunit beta-1	Ap3b1	7	11	1,681449316
**Q9D0M3**	Cytochrome c1, heme protein, mitochondrial	Cyc1	9	38,2	1,236597124
**O08547**	Vesicle-trafficking protein SEC22b	Sec22b	9	34	1,163500498
**P97762**	Retinitis pigmentosa 9 protein homolog	rp9	9	33,8	1,048972924
**A2AJT4**	Arginine/serine-rich protein PNISR	Pnisr	8	9,6	13,34151882
**Q99ME9**	Nucleolar GTP-binding protein 1	Gtpbp4	8	16,1	12,49832567
**Q6P9S0**	MTSS1-like protein	Mtss1l	8	21	12,2625647
**Q9CSP9**	Tetratricopeptide repeat protein 14	Ttc14	8	12,7	4,261251796
**Q9ESU6**	Bromodomain-containing protein 4	Brd4	8	11,5	2,78047406
**Q6PDM2; Q9D0B0**	Serine/arginine-rich splicing factor 1	Srsf1	8	37,1	2,589945724
**Q61543**	Golgi apparatus protein 1	Glg1	8	11,4	2,578721452
**Q3UMQ8**	H/ACA ribonucleoprotein complex non-core subunit NAF1	Naf1	8	31,1	2,376996492
**Q9CR80**	Protein FAM32A	Fam32a	8	48,2	2,302596004
**Q921I2**	Kelch domain-containing protein 4	Klhdc4	8	18,5	2,099199111
**Q8BMA6**	Signal recognition particle subunit SRP68	Srp68	8	19,7	1,820212035
**O55128**	Histone deacetylase complex subunit SAP18	Sap18	8	45,1	1,807422151
**Q9ESW4**	Acylglycerol kinase, mitochondrial	Agk	8	26,8	1,664031769
**P47856**	Glutamine-fructose-6-phosphate aminotransferase [isomerizing] 1	Gfpt1	7	17,8	1,516397856
**Q9D8X2**	Coiled-coil domain-containing protein 124	Ccdc124	8	35,5	1,458399982
**Q8VEM8**	Phosphate carrier protein, mitochondrial	Slc25a3	8	26,6	1,310700537
**Q99N93**	39S ribosomal protein L16, mitochondrial	Mrpl16	8	31,9	1,189945134
**Q8BMJ3;Q60872;J3QNT6;Q3UTA4;Q3TQZ4;J3QQ02;J3QPI8;J3QP87;A0**	Eukaryotic translation initiation factor 1A, X-chromosomal; Eukaryotic translation initiation factor 1A	Eif1ax;Eif1a	8	69,4	1,156827885

**A1Y7VNG9;A0A1Y7VLT7;A0A1Y7VK80;A0A1Y7VJE9;Q8BX20;Q3UT53;J3QMW5**					
**P24369**	Peptidyl-prolyl cis-trans isomerase B	Ppib	8	41,2	1,096503284
**Q91VA7**	Isocitrate dehydrogenase [NAD] subunit, mitochondrial	Idh3b	8	27,6	1,083705815
**Q3UY34**	Uncharacterized protein C12orf43 homolog		7	49,6	12,4655409
**Q91X97; P84075**	Neurocalcin-delta	Ncald	5	34,2	12,3343571
**Q8BG79**	CWF19-like protein 2	Cwf19l2	7	9,5	12,14602764
**P62874; P29387; Q61011**	Guanine nucleotide-binding protein G(I)/G(S)/G(T) subunit beta-1	Gnb1	2	26,5	10,23876298
**Q6GQS1**	Calcium-binding mitochondrial carrier protein SCaMC-3	Slc25a23	7	21,2	4,438015112
**Q9DBF7**	Pre-mRNA-splicing factor CWC25 homolog	Cwc25	7	16,1	2,926923208
**Q9D710**	Thioredoxin-related transmembrane protein 2	Tmx2	7	30,5	2,414742526
**Q80X85**	28S Ribosomal protein S7, mitochondrial	Mrps7	7	42,1	2,180286639
**Q9CVI2**	Protein FAM133B	Fam133b	7	27,8	2,060257099
**P83887; Q8VCK3**	Tubulin gamma-1 chain; Tubulin gamma-2 chain	Tubg1; Tubg2	7	26,8	2,059437414
**Q9D328**	Transmembrane protein 35	Tmem35	7	23,4	1,643186899
**P97807**	Fumarate hydratase, mitochondrial	Fh	7	22,9	1,520132022
**Q8VHE0**	Translocation protein SEC63 homolog	Sec63	7	12,6	1,281150847
**P06745; CON__Q3ZBD7**	Glucose-6-phosphate isomerase	Gpi	7	23,1	1,217423794
**Q9EPL8**	Importin-7	Ipo7	7	7,5	1,085930743
**Q9R087**	Glypican-6; Secreted glypican-6	Gpc6	7	20,9	1,073599996
**P61222**	ATP-binding cassette sub-family E member 1	Abce1	7	15,4	1,070229698
**Q8R323**	Replication factor C subunit 3	Rfc3	7	21,9	1,025277644

**P08752; B2RSH2; Q9DC51; P20612; Q3V3I2; P50149; P18872; Q8CGK7**	Guanine nucleotide-binding protein G(i) subunit alpha-2; Guanine nucleotide-binding protein G(i) subunit alpha-1	Gnai2; Gnai1	6	23,9	1,019123775
**Q9CWX9**	Probable ATP-dependent RNA helicase DDX47	Ddx47	6	20,2	12,48728736
**Q8BGH4**	Receptor expression-enhancing protein 1	Reep1	6	29,9	12,1365426
**Q8BU14**	Translocation protein SEC62	Sec62	6	12,1	11,92224989
**Q8BYC6**	Serine/threonine-protein kinase TAO3	Taok3	6	10,1	11,49255424
**Q9CS84**	Neurexin-1	Nrxn1	4	5	10,84180019
**O88665**	Bromodomain-containing protein 7	Brd7	6	15,5	4,686056343
**Q8C4U3**	Secreted frizzled-related protein 1	Sfrp1	6	27,1	4,181750437
**O88844**	Isocitrate dehydrogenase [NADP] cytoplasmic	Idh1	6	21,3	3,675409449
**Q9D0K1**	Peroxisomal membrane protein PEX13	Pex13	6	21,5	3,315077529
**Q8BHF7**	CDP-diacylglycerol-glycerol-3-phosphate 3-phosphatidyltransferase, mitochondrial	Pgs1	6	14,8	2,865103453
**Q922B2**	Aspartate-tRNA ligase, cytoplasmic	Dars	6	16,2	2,829998016
**Q8BVY0**	Ribosomal L1 domain-containing protein 1	Rsl1d1	6	13,1	2,635821123
**P58059**	28S Ribosomal protein S21, mitochondrial	Mrps21	6	65,5	2,509061984
**O88455**	7-Dehydrocholesterol reductase	Dhcr7	6	15,9	2,272261634
**Q9D023**	Mitochondrial pyruvate carrier 2	Mpc2	6	47,2	2,269749605
**Q61070**	Etoposide-induced protein 2.4	Ei24	6	15,6	2,159615199
**Q8C2Q3; J3QN51; B0LM42; F7BGR7**	RNA-binding protein 14	Rbm14	6	13	1,991782654
**Q922J9**	Fatty acyl-CoA reductase 1	Far1	6	15,5	1,870304568
**Q9CPW7**	Zinc-finger matrin-type protein 2	Zmat2	6	25,6	1,866563441
**Q9R1C7**	Pre-mRNA-processing factor 40 homolog A	Prpf40a	6	7,8	1,818505391

**Q5XJY5**	Coatomer subunit delta	Arcn1	6	17,8	1,804023843
**Q80XU3**	Nuclear ubiquitous casein and cyclin-dependent kinase substrate 1	Nucks1	6	24,8	1,702846216
**Q6P3B9**	Putative ribosome-binding factor A, mitochondrial	Rbfa	6	25,4	1,5557011
**Q60931**	Voltage-dependent anion-selective channel protein 3	Vdac3	6	21,6	1,552062861
**Q9QYF1**	Retinol dehydrogenase 11	Rdh11	6	23,1	1,441371215
**Q9CQU3**	Protein RER1	Rer1	6	37,8	1,345395073
**Q9D0F4; Q5SZT7**	NF-kappa-B-activating protein	Nkap;Nkapl	6	16,1	1,295856175
**P83882; A0A2I3BPG9; A0A0A6YW33**	60S ribosomal protein L36a	Rpl36a;Gm6525	6	27,4	1,288763667
**P51880**	Fatty acid-binding protein, brain	Fabp7	6	43,9	1,173321545
**Q8BG32**	26S proteasome non-ATPase regulatory subunit 11	Psmd11	6	17,3	1,068507925
**Q9CQ69**	Cytochrome b-c1 complex subunit 8	Uqcrq	6	61	1,034471864
**Q9EPJ9**	ADP-ribosylation factor GTPase-activating protein 1	Arfgap1	5	18,6	11,69252833
**E9Q4F7**		Ankrd11	5	2,4	11,63058567
**Q9DBU6**	Serine/arginine-related protein 53	Rsrc1	5	22,8	11,59781979
**P97432**	Next to BRCA1 gene 1 protein	Nbr1	5	6,4	11,51412226
**Q5HZI9**	Solute carrier family 25 member 51	Slc25a51	5	19,1	11,40482225
**Q07235**	Glia-derived nexin	Serpine2	5	14,6	11,26426665
**Q922R8**	Protein disulfide-isomerase A6	Pdia6	5	23,2	11,23302043
**P60122**	RuvB-like 1	Ruvbl1	5	16,9	10,98598421
**E9QAT4**		Sec16a	5	3,3	10,95441444
**Q69ZS7**	HBS1-like protein	Hbs1l	5	9,5	10,94097422
**Q5U3K5**	Rab-like protein 6	Rabl6	5	8,8	10,90726625
**P26039; Q71LX4**	Talin-1	Tln1	5	3,3	9,229179319
**Q3TKT4**	Transcription activator BRG1	Smarca4	2	3,8	8,424754167
**Q9D517**	1-acyl-sn-glycerol-3-phosphate acyltransferase gamma	Agpat3	5	13,6	4,103219273
**Q9DCN2**	NADH-cytochrome b5 reductase 3; NADH-cytochrome b5 reductase 3 membrane-bound form; NADH-cytochrome b5 reductase 3 soluble form	Cyb5r3	5	22,3	3,947461779

**P58021**	Transmembrane 9 superfamily member 2	Tm9sf2	5	9,2	3,895936638
**Q61598; P50396**	Rab GDP dissociation inhibitor beta	Gdi2	5	18,7	3,464047134
**Q8BP92**	Reticulocalbin-2	Rcn2	5	19,1	3,335080677
**Q7TN98; Q812E0; Q7TN99**	Cytoplasmic polyadenylation element-binding protein 4	Cpeb4	5	9,5	3,271948918
**F6TVX7; Q8BSF4**	Phosphatidylserine decarboxylase proenzyme; Phosphatidylserine decarboxylase alpha chain; Phosphatidylserine decarboxylase beta chain	Gm20671;Pisd	5	11,8	3,182982998
**Q8BLH7**	HIRA-interacting protein 3	Hirip3	5	12,6	3,084734742
**Q5HZG4**	Transcription initiation factor TFIID subunit 3	Taf3	5	7,7	2,6269542
**Q8BKI2**	Trinucleotide repeat-containing gene 6B protein	Tnrc6b	5	5	2,532729198
**Q8R3N1**	Nucleolar protein 14	Nop14	5	7,4	2,513148161
**Q3THK3**	General transcription factor IIF subunit 1	Gtf2f1	5	13,6	2,465057564
**Q8R2M2**	Deoxynucleotidyltransferase terminal-interacting protein 2	Dnttip2	5	8,3	2,433993768
**Q8BP47**	Asparagine--tRNA ligase, cytoplasmic	Nars	5	10,9	2,42619632
**P70296**	Phosphatidylethanolamine-binding protein 1; Hippocampal cholinergic neurostimulating peptide	Pebp1	5	44,9	2,366572398
**Q99LC5**	Electron transfer flavoprotein subunit alpha, mitochondrial	Etfa	5	22,2	2,227551819
**Q91XC9**	Peroxisomal membrane protein PEX16	Pex16	5	18,5	2,164524774
**Q80VA0**	N-Acetylgalactosaminyltransferase 7	Galnt7	5	13,4	2,036034347
**Q3UUQ7**	GPI inositol-deacylase	Pgap1	5	7,4	1,745372182
**Q6ZPZ3**	Zinc-finger CCCH domain-containing protein 4	Zc3h4	5	8,5	1,694537053
**E9PUQ3**		AU019823	5	20,2	1,544314993
**Q99LY9**	NADH dehydrogenase [ubiquinone] iron-sulfur protein 5; NADH dehydrogenase [ubiquinone] iron-sulfur protein 5, N-terminally processed	Ndufs5	5	28,3	1,34899142

**Q0VG62**	Uncharacterized protein C8orf59 homolog		5	48,5	1,285845151
**Q9D1F0**		Cxx1a	5	33,6	1,253276192
**Q9R1J0**	Sterol-4-alpha-carboxylate 3-dehydrogenase, decarboxylating	Nsdhl	5	17,4	1,248456403
**Q80UM3; Q9DBB4**	N-Alpha-acetyltransferase 15, NatA auxiliary subunit	Naa15	5	6,1	1,222759739
**Q4QQM4**	Tumor protein p53-inducible protein 11	Trp53i11	5	24,3	1,222004418
**Q8VCL2**	Protein SCO2 homolog, mitochondrial	Sco2	5	32,2	1,129699616
**P35278; P61021**	Ras-related protein Rab-5C	Rab5c	3	27,3	1,087536163
**Q64521**	Glycerol-3-phosphate dehydrogenase, mitochondrial	Gpd2	5	7,6	1,06656929
**Q9CPQ8**	ATP synthase subunit g, mitochondrial	Atp5l	5	47,6	1,047765484
**P62849**	40S ribosomal protein S24	Rps24	5	30,1	1,00757959
**Q99J99**	3-mercaptopyruvate sulfurtransferase	Mpst	4	23,6	11,93129155
**Q8VDP2**	UPF0428 protein CXorf56 homolog		4	22,1	11,80207202
**Q9CQV1**	Mitochondrial import inner membrane translocase subunit TIM16	Pam16	4	49,6	11,76109408
**Q8CI11**	Guanine nucleotide-binding protein-like 3	Gnl3	4	13,2	11,0146482
**Q8VI75**	Importin-4	Ipo4	4	4,3	10,99604973
**Q3TDQ1**	Dolichyl-diphosphooligosaccharide--protein glycosyltransferase subunit STT3B	Stt3b	4	7,7	10,97935374
**Q8C163**	Nuclease EXOG, mitochondrial	Exog	4	16,6	10,84509796
**Q920Q6**	RNA-binding protein Musashi homolog 2	Msi2	4	13,9	10,84399954
**Q02257**	Junction plakoglobin	Jup	4	7,7	10,74155125
**P35486**	Pyruvate dehydrogenase E1 component subunit alpha, somatic form, mitochondrial	Pdha1	4	17,2	10,70856662
**Q7TQ95**	Protein lunapark	Lnp	4	13,2	10,69939894
**Q3TFK5**	G patch domain-containing protein 4	Gpatch4	4	15,4	10,64015454
**Q6ZPL9**	ATP-dependent RNA helicase DDX55	Ddx55	4	10,3	10,6319955

**E9Q3G8**		Nup153	4	4,6	10,56814614
**Q6PB66**	Leucine-rich PPR motif-containing protein, mitochondrial	Lrpprc	4	5	10,46454575
**Q9DAW9**	Calponin-3	Cnn3	4	17,3	10,25903704
**Q8CJG0; Q8CJF9**	Protein argonaute-2	Ago2	4	6,9	10,11022228
**Q61749**	Translation initiation factor eIF-2B subunit delta	Eif2b4	4	13,2	10,09539702
**Q64514**	Tripeptidyl-peptidase 2	Tpp2	4	3,6	10,09011242
**A0A0J9YUD5**			4	2,6	9,895014757
**E9Q7X7**		Nrxn2	4	5,3	9,783996778
**Q9R1T2**	SUMO-activating enzyme subunit 1; SUMO-activating enzyme subunit 1, N-terminally processed	Sae1	4	19,7	9,686500527
**Q9DBG3; O35643**	AP-2 complex subunit beta	Ap2b1	4	5,8	9,643459394
**Q9EQP2**	EH domain-containing protein 4	Ehd4	3	8,1	9,287158278
**Q99NB9**	Splicing factor 3B subunit 1	Sf3b1	4	4,7	9,074328814
**Q9D0M1**	Phosphoribosyl pyrophosphate synthase-associated protein 1	Prpsap1	4	15,4	8,840400288
**Q8C6B9**	Active regulator of SIRT1	Rps19bp1	4	42	4,693739576
**Q9CQ54**	NADH dehydrogenase [ubiquinone] 1 subunit C2	Ndufc2	4	20,8	4,53900926
**Q921V5**	Alpha-1,6-mannosyl-glycoprotein 2-beta-N-acetylglucosaminyltransferase	Mgat2	4	13,3	3,180016519
**P61620; Q9JLR1**	Protein transport protein Sec61 subunit alpha isoform 1	Sec61a1	4	10,9	2,774104687
**Q8C1S0**	Mediator of RNA polymerase II transcription subunit 19	Med19	4	31,6	2,677165983
**Q8BTU1**	Cilia- and flagella-associated protein 20	Cfap20	4	25,4	2,491967475
**P19258**	Protein Mpv17	Mpv17	4	25	2,203934832
**Q924Z4**	Ceramide synthase 2	Cers2	4	11,3	2,128952547
**P52293**	Importin subunit alpha-1	Kpna2	4	11,7	2,123164155
**Q01721**	Growth arrest-specific protein 1	Gas1	4	25,4	2,025649586
**Q8R0A0**	General transcription factor IIF subunit 2	Gtf2f2	4	22,5	1,988784564
**P19783**	Cytochrome c oxidase subunit 4 isoform 1, mitochondrial	Cox4i1	4	30,8	1,890080375

**Q9CXU9; P48024**	Eukaryotic translation initiation factor 1b; Eukaryotic translation initiation factor 1	Eif1b;Eif1	4	36,3	1,857085932
**Q9ERS2**	NADH dehydrogenase [ubiquinone] 1 alpha subcomplex subunit 13	Ndufa13	4	29,9	1,648583853
**Q91VU0**	Protein FAM3C	Fam3c	4	17,6	1,568442268
**A0A0J9YTR2**			4	38,9	1,521739224
**Q60714**	Long-chain fatty acid transport protein 1	Slc27a1	4	11	1,515725525
**Q9JIG8; A0A140LJ36**	PRA1 family protein 2	Praf2	4	20,8	1,443652639
**P52912**	Nucleolysin TIA-1	Tia1	4	22,8	1,358228368
**Q9JKW0**	ADP-ribosylation factor-like protein 6-interacting protein 1	Arl6ip1	4	16,7	1,189137625
**Q99PL7; P13516; P13011; Q6T707**	Acyl-CoA desaturase 1; Acyl-CoA desaturase 2	Scd3;Scd1;Scd2;Scd4	4	7,2	1,164236982
**Q8R127**	Saccharopine dehydrogenase-like oxidoreductase	Sccpdh	4	10	1,141404163
**Q6PCM2**	Integrator complex subunit 6	Ints6	4	5,3	1,047590042
**P70213**	Friend virus susceptibility protein 1	Fv1	4	12,4	1,034038461
**Q9D6K8**	FUN14 domain-containing protein 2	Fundc2	3	23,2	12,81051139
**Q8VD00**	Transmembrane protein 97	Tmem97	3	15,9	12,45249866
**Q80TJ7; Q9WTU0**	Histone lysine demethylase PHF8	Phf8	3	4,8	11,94251451
**O35988**	Syndecan-4	Sdc4	3	19,7	11,93520189
**P63330 ;P62715**	Serine/threonine-protein phosphatase 2A catalytic subunit alpha isoform; Serine/threonine-protein phosphatase 2A catalytic subunit beta isoform	Ppp2ca; Ppp2cb	3	16,2	11,33594857
**Q8C6C7**	Protein FAM204A	Fam204a	3	35,6	11,21377254
**Q9ESX4**	Nucleolar protein of 40 kDa	Zcchc17	3	13,3	11,08766173
**Q4V9W2**	Protein SREK1IP1	Srek1ip1	3	20,9	11,02292259
**Q3UMM4**	Cyclin-dependent kinase 10	Cdk10	3	9,7	10,95963976
**Q91Z92**	Beta-1,3-galactosyltransferase 6	B3galt6	3	12,6	10,94434603
**Q9CRB2**	H/ACA ribonucleoprotein complex subunit 2	Nhp2	3	28,1	10,89875253
**P35979**	60S ribosomal protein L12	Rpl12	3	28,5	10,88218449

**Q9JJE4**	Progestin and adipoQ receptor family member 4	Paqr4	3	11,7	10,87950667
**Q8R5J9**	PRA1 family protein 3	Arl6ip5	3	19,7	10,85992297
**P83510**	Traf2 and NCK-interacting protein kinase	Tnik	3	6	10,60529431
**Q9D5T0**	ATPase family AAA domain-containing protein 1	Atad1	3	13,6	10,60334816
**Q99N85**	28S ribosomal protein S18a, mitochondrial	Mrps18a	3	23	10,55276515
**O35295; P42669**	Transcriptional activator protein Pur-beta	Purb	3	17,9	10,51796459
**Q9DA19**	Corepressor interacting with RBPJ 1	Cir1	3	11,3	10,47482133
**O70480**	Vesicle-associated membrane protein 4	Vamp4	3	23,4	10,4708622
**Q9WU56**	tRNA pseudouridine synthase A, mitochondrial	Pus1	3	10,6	10,43858396
**Q8VCS3**	Glycosaminoglycan xylosylkinase	Fam20b	3	11	10,39446269
**Q8K4F6**	Probable 28S rRNA (cytosine-C(5))-methyltransferase	Nsun5	3	8,6	10,38963134
**P16330**	2,3-Cyclic-nucleotide 3-phosphodiesterase	Cnp	3	12,1	10,38661698
**Q6PHQ8**	N-alpha-acetyltransferase 35, NatC auxiliary subunit	Naa35	3	3,3	10,34340782
**Q60598**	Src substrate cortactin	Cttn	3	9,9	10,3178648
**Q9D0D4**	Probable dimethyladenosine transferase	Dimt1	3	14,7	10,3089078
**Q9DBJ1; O70250**	Phosphoglycerate mutase 1	Pgam1	3	20,5	10,29404631
**P48432; Q04892; Q811W0; P53784; P53783; Q05738**	Transcription factor SOX-2	Sox2	3	13,5	10,28863541
**Q9Z1R4**	Uncharacterized protein C6orf47 homolog	D17h6s53e	3	12,6	10,19229281
**Q9DBM1**	G patch domain-containing protein 1	Gpatch1	3	5,7	10,1323712
**E9PWG6**		Ncapg	3	4,3	10,1048608
**Q8BH24**	Transmembrane 9 superfamily member 4	Tm9sf4	3	6,2	10,10079373

**Q9CXI5**	Mesencephalic astrocyte-derived neurotrophic factor	Manf	3	19	9,962809268
**Q3U4G3**	Xyloside xylosyltransferase 1	Xxylt1	3	7,9	9,961739073
**Q921H8; Q8VCH0**	3-Ketoacyl-CoA thiolase A, peroxisomal; 3-Ketoacyl-CoA thiolase B, peroxisomal	Acaa1a; Acaa1b	3	12,3	9,930486027
**Q91XV3**	Brain acid soluble protein 1	Basp1	3	25,7	9,724820777
**Q69ZC8**	GPALPP motifs-containing protein 1	Gpalpp1	3	11	9,721834024
**Q9QYE6**	Golgin subfamily A member 5	Golga5	3	6,4	9,717950543
**Q9R0H0**	Peroxisomal acyl-coenzyme A oxidase 1	Acox1	3	6,8	9,587852508
**A2ASQ1**	Agrin; Agrin N-terminal 110 kDa subunit; Agrin C-terminal 110 kDa subunit; Agrin C-terminal 90 kDa fragment; Agrin C-terminal 22 kDa fragment	Agrn	3	2,8	9,579202781
**A2ACQ1**		Dhx35	3	6,7	9,532044168
**Q9DBR1**	5-3 Exoribonuclease 2	Xrn2	3	4,2	9,439934746
**Q7TNL5**		Ppp2r5d	3	6,2	9,322491538
**P58501**	PAX3- and PAX7-binding protein 1	Paxbp1	3	3,9	9,25074845
**Q921N6**	Probable ATP-dependent RNA helicase DDX27	Ddx27	3	4,7	9,062909607
**Q8VI33; Q6NZA9**	Transcription initiation factor TFIID subunit 9	Taf9	3	21,2	8,748427688
**P70372**	ELAV-like protein 1	Elavl1	3	9,5	8,700023496
**Q8BI72**	CDKN2A-interacting protein	Cdkn2aip	3	9,1	8,584248491
**P13439**	Uridine 5-monophosphate synthase; Orotate phosphoribosyltransferase; Orotidine 5-phosphate decarboxylase	Umps	3	9,1	8,286557762
**P46471**	26S protease regulatory subunit 7	Psmc2	3	9,7	8,197216693
**Q8VDF2; Q7TMI3**	E3 ubiquitin-protein ligase UHRF1	Uhrf1	3	4,7	8,034578897
**Q9EPE9**	Manganese-transporting ATPase 13A1	Atp13a1	3	3,3	7,272583133
**P99028**	Cytochrome b-c1 complex subunit 6, mitochondrial	Uqcrh	3	39,3	3,034816542
**Q91XL3**	UDP-glucuronic acid decarboxylase 1	Uxs1	3	12,6	2,833976492
**Q8R1I1**	Cytochrome b-c1 complex subunit 9	Uqcr10	3	39,1	2,334574151

**Q6Y7W8**	PERQ amino acid-rich with GYF domain-containing protein 2	Gigyf2	3	3,1	2,252557603
**O35887**	Calumenin	Calu	3	12,7	2,140419788
**Q6PGH1**	Protein BUD31 homolog	Bud31	3	21,5	2,106633893
**Q9ERY9**	Probable ergosterol biosynthetic protein 28	ORF11	3	22,9	2,071570855
**D3Z4I3; Q62176**	RNA-binding protein 24; RNA-binding protein 38	Rbm24;Rbm 38	3	16,1	1,956632027
**Q8C1Q6**	Small integral membrane protein 4	Smim4	3	30	1,81465017
**Q5XKN4**	Protein jagunal homolog 1	Jagn1	3	13,7	1,765369515
**Q8VCH6**	Delta(24)-sterol reductase	Dhcr24	3	5,6	1,718954272
**Q9D8M4**	60S Ribosomal protein L7-like 1	Rpl7l1	3	15,9	1,587315747
**Q91ZN5**	Adenosine 3-phospho 5-phosphosulfate transporter 1	Slc35b2	3	8,1	1,494623142
**Q9JKN5; Q6PFG8**	Oligodendrocyte transcription factor 1	Olig1	2	13,1	1,48685709
**O54825**	Bystin	Bysl	3	6,7	1,466149169
**Q4FK66**	Pre-mRNA-splicing factor 38A	Prpf38a	3	11,5	1,188896617
**Q99JI6; P62835**	Ras-related protein Rap-1b; Ras-related protein Rap-1A	Rap1b;Rap1 a	3	17,9	1,110584115
**Q66GT5**	Phosphatidylglycerophosphatase and protein-tyrosine phosphatase 1	Ptpmt1	3	19,7	1,027166362
**P10107**	Annexin A1	Anxa1	3	4,9	1,024739451
**A0A1W2P7S8; Q6A068;A0A1 W2P855;A0A1 W2P7Z8;A0A1 W2P7H4;A0A 1W2P6Q8**	Cell division cycle 5-like protein	Cdc5l	3	5	1,012596516
**Q8BGX2**	Uncharacterized protein C19orf52 homolog		3	17,3	1,002431851
**Q8K072**	Receptor expression-enhancing protein 4	Reep4	2	3,1	12,50695216
**Q6A028**	Switch-associated protein 70	Swap70	2	1,7	11,30714387
**Q9WUK2**	Eukaryotic translation initiation factor 4H	Eif4h	2	12,9	11,29462075
**Q9Z2N8**	Actin-like protein 6A	Actl6a	2	10,3	11,20951429
**Q8K363**	ATP-dependent RNA helicase DDX18	Ddx18	2	3,5	11,1866714
**D3Z3N4**		Hnrnph3	2	8,4	11,15690471
**Q3UN02**	Lysocardiolipin acyltransferase 1	Lclat1	2	9	11,08494123

**P01863; P01865; A0A0A6YY53; F6TQW2; P01864**	Ig gamma-2A chain C region, A allele; Ig gamma-2A chain C region, membrane-bound form; Ig gamma-2A chain C region secreted form	Ighg;Igh-1a;Ighg2c	2	10	11,07434219
**Q9R0N7**	Synaptotagmin-7	Syt7	2	10,7	10,90425828
**O09005**	Sphingolipid delta(4)-desaturase DES1	Degs1	2	10,5	10,84729229
**Q9JIX0**	Transcription and mRNA export factor ENY2	Eny2	2	25,7	10,82472013
**Q99PG2**	Opioid growth factor receptor	Ogfr	2	4,3	10,68176549
**Q60875**	Rho guanine nucleotide exchange factor 2	Arhgef2	2	3,5	10,55478069
**Q9CQF8**	Ribosomal protein 63, mitochondrial	Mrpl57	2	13,7	10,52894242
**Q9JJ61; Q8BVG5**	Polypeptide N-acetylgalactosaminyltransferase 16; Polypeptide N-acetylgalactosaminyltransferase 14	Galnt16;Galnt14	2	5,4	10,50898318
**P42925**	Peroxisomal membrane protein 2	Pxmp2	2	11,3	10,42143371
**Q8BK08**	Transmembrane protein 11, mitochondrial	Tmem11	2	13,2	10,41500228
**Q6TEK5**	Vitamin K epoxide reductase complex subunit 1-like protein 1	Vkorc1l1	2	11,4	10,36763316
**Q8R2L5**	28S Ribosomal protein S18c, mitochondrial	Mrps18c	2	23,8	10,35060774
**P62880**	Guanine nucleotide-binding protein G(I)/G(S)/G(T) subunit beta-2	Gnb2	2	28,5	10,33516701
**Q9CX30**	Protein YIF1B	Yif1b	2	10,9	10,3254179
**Q9D880**	Mitochondrial import inner membrane translocase subunit TIM50	Timm50	2	7,9	10,31015832
**P21981**	Protein-glutamine gamma-glutamyltransferase 2	Tgm2	2	4,5	10,28655776
**Q8JZX4**	Splicing factor 45	Rbm17	2	6,4	10,27961058
**Q8VDL4**	ADP-dependent glucokinase	Adpgk	2	6	10,2769386
**Q9JHN8**	Serine/threonine-protein kinase 19	Stk19	2	9,1	10,27286302
**Q9Z2W1; Q99JT2; Q99KH8**	Serine/threonine-protein kinase 25; Serine/threonine-protein kinase 26; Serine/threonine-protein kinase 24; Serine/threonine-protein kinase 24 35 kDa subunit; Serine/threonine-protein kinase 24 12 kDa subunit	Stk25; Stk26; Stk24	2	6,6	10,17130192

**Q9JIH2**	Nuclear pore complex protein Nup50	Nup50	2	6,9	10,11309098
**O88271**	Craniofacial development protein 1	Cfdp1	2	12,5	10,11113567
**Q6ZQI3**	Malectin	Mlec	2	8,6	10,07921809
**P99027**	60S Acidic ribosomal protein P2	Rplp2	2	42,6	10,02652344
**P35601**	Replication factor C subunit 1	Rfc1	2	2,8	10,00112667
**O70579**	Peroxisomal membrane protein PMP34	Slc25a17	2	7,5	9,999718196
**Q6PGG6**	Guanine nucleotide-binding protein-like 3-like protein	Gnl3l	2	5,5	9,9893945
**Q9CR59**	Growth arrest and DNA damage-inducible proteins-interacting protein 1	Gadd45gip1	2	9,9	9,981567282
**Q9CRD2**	ER membrane protein complex subunit 2	Emc2	2	8,4	9,980139578
**Q3TKY6**	Peptidyl-prolyl cis-trans isomerase CWC27 homolog	Cwc27	2	5,3	9,973697366
**O35083**	1-Acyl-sn-glycerol-3-phosphate acyltransferase alpha	Agpat1	2	15,4	9,954618004
**Q8BFV2**	PCI domain-containing protein 2	Pcid2	2	7	9,94966624
**P61161**	Actin-related protein 2	Actr2	2	6,6	9,9408568
**Q8BSL7; P84078; P61205**	ADP-ribosylation factor 2; ADP-ribosylation factor 1; ADP-ribosylation factor 3	Arf2;Arf1;Arf3	2	34,3	9,932657681
**Q9WTQ8**	Mitochondrial import inner membrane translocase subunit Tim23	Timm23	2	12,9	9,92982058
**Q61166**	Microtubule-associated protein RP/EB family member 1	Mapre1	2	14,2	9,891996295
**Q8BHS6**	Armadillo repeat-containing X-linked protein 3	Armcx3	2	7,4	9,87774425
**Q9CWS0**	N(G),N(G)-dimethylarginine dimethylaminohydrolase 1	Ddah1	2	12,6	9,873536393
**Q922Q1**	Mitochondrial amidoxime reducing component 2	02-Mar	2	7,4	9,868591088
**Q9ET30**	Transmembrane 9 superfamily member 3	Tm9sf3	2	3,6	9,863628773
**Q8C4Q6**	Axin interactor, dorsalization-associated protein	Aida	2	10,5	9,830340901
**Q80YV2**	Nuclear-interacting partner of ALK	Zc3hc1	2	6,4	9,822554898
**Q6P1J0**	Glycoprotein endo-alpha-1,2-mannosidase-like protein	Maneal	2	7,1	9,808545942

**Q8C7X2**	ER membrane protein complex subunit 1	Emc1	2	4,7	9,789908534
**O08529**	Calpain-2 catalytic subunit	Capn2	2	3,7	9,782965457
**Q9CQZ6**	NADH dehydrogenase [ubiquinone] 1 beta subcomplex subunit 3	Ndufb3	2	18,3	9,767605145
**Q8CB44**	GRAM domain-containing protein 4	Gramd4	2	5,5	9,705079392
**P24270**	Catalase	Cat	2	7,8	9,69115027
**Q8K4R9**	Disks large-associated protein 5	Dlgap5	2	2,2	9,68187088
**Q91VT4**	Carbonyl reductase family member 4	Cbr4	2	11	9,672036328
**Q8CIL4**	Uncharacterized protein C1orf131 homolog		2	8,9	9,659585674
**Q8BXA5**	Cleft lip and palate transmembrane protein 1-like protein	Clptm1l	2	6,3	9,654206378
**Q99KV1**	DnaJ homolog subfamily B member 11	Dnajb11	2	8,7	9,640118378
**Q8K0T0**	Reticulon-1	Rtn1	2	2,7	9,632468172
**Q9CXG3**	Peptidyl-prolyl cis-trans isomerase-like 4	Ppil4	2	7,7	9,616199614
**Q9DCJ7**	Aurora kinase A-interacting protein	Aurkaip1	2	7	9,5413677
**Q922Q2**	Serine/threonine-protein kinase RIO1	Riok1	2	4,8	9,531283978
**O35493; P22518**	Dual specificity protein kinase CLK4; Dual specificity protein kinase CLK1	Clk4;Clk1	2	6	9,44819892
**Q9R099**	Transducin beta-like protein 2	Tbl2	2	5,7	9,447971719
**Q8VBT0**	Thioredoxin-related transmembrane protein 1	Tmx1	2	9	9,445056243
**Q80W03; Q66JW3; Q8BU11**	TOX high mobility group box family member 3; Thymocyte selection-associated high mobility group box protein TOX; TOX high mobility group box family member 4	Tox3;Tox;Tox4	2	4,3	9,417029691
**Q3UHX9**	Putative methyltransferase C9orf114 homolog	D2Wsu81e	2	11,9	9,375669387
**Q8BNI4**	Derlin-2	Derl2	2	17,2	9,373604714
**Q8VBZ3**	Cleft lip and palate transmembrane protein 1 homolog	Clptm1	2	7,5	9,362382216
**P70404**	Isocitrate dehydrogenase [NAD] subunit gamma 1, mitochondrial	Idh3g	2	9,2	9,328450541
**Q06185**	ATP synthase subunit e, mitochondrial	Atp5i	2	32,4	9,279842694

**P30658**	Chromobox protein homolog 2	Cbx2	2	5,6	9,277682605
**Q00PI9**	Heterogeneous nuclear ribonucleoprotein U-like protein 2	Hnrnpul2	2	4,4	9,270902649
**Q60864**	Stress-induced-phosphoprotein 1	Stip1	2	7,6	9,23935985
**Q8R3S6**	Exocyst complex component 1	Exoc1	2	3	9,23570313
**Q9CXW3**	Calcyclin-binding protein	Cacybp	2	13,5	9,156866799
**Q61595**	Kinectin	Ktn1	2	2	9,14407187
**Q9QUR6**	Prolyl endopeptidase	Prep	2	4,2	9,124095457
**Q922V4**	Pleiotropic regulator 1	Plrg1	2	6,8	9,096135325
**P29758**	Ornithine aminotransferase, mitochondrial	Oat	2	6,6	9,094526396
**Q8VE70**	Programmed cell death protein 10	Pdcd10	2	10,8	9,08976825
**P68404**	Protein kinase C beta type	Prkcb	2	2,5	9,075211711
**Q9WTR5**	Cadherin-13	Cdh13	2	3,6	9,047069367
**Q9QYB1**	Chloride intracellular channel protein 4	Clic4	2	15,8	9,041823396
**Q9CW46**	Ribonucleoprotein PTB-binding 1	Raver1	2	4,4	9,024973407
**P34022**	Ran-specific GTPase-activating protein	Ranbp1	2	16,7	9,014159989
**Q8BGQ7**	Alanine--tRNA ligase, cytoplasmic	Aars	2	2,9	9,01150682
**P62492; P46638**	Ras-related protein Rab-11A; Ras-related protein Rab-11B	Rab11a; Rab11b	2	11,1	8,996925364
**Q91VE0**	Long-chain fatty acid transport protein 4	Slc27a4	2	5,1	8,976019878
**Q5RJG1**	Nucleolar protein 10	Nol10	2	3,2	8,958233692
**E9QAP7; G5E8Z2**	Transcription initiation factor TFIID subunit 4B	Taf4a; Taf4b	2	4,1	8,929110432
**Q9R1B9**	Slit homolog 2 protein; Slit homolog 2 protein N-product; Slit homolog 2 protein C-product	Slit2	2	1,5	8,916655776
**Q99LR1**	Monoacylglycerol lipase ABHD12	Abhd12	2	4,3	8,832795097
**Q99JT5**	Lysine-rich coiled-coil protein 1	Krcc1	2	10,5	8,814262009
**Q8CG48**	Structural maintenance of chromosomes protein 2	Smc2	2	2	8,810410969
**Q9CRG1**	Transmembrane 7 superfamily member 3	Tm7sf3	2	5	8,795877317
**Q9JJK2**	LanC-like protein 2	Lancl2	2	7,1	8,78260514
**P27601**	Guanine nucleotide-binding protein subunit alpha-13	Gna13	2	5,3	8,77603817
**E9Q9Q2**		R3hdm1	2	2,4	8,74929961
**Q8K205**		Pop1	2	2,8	8,73369328

**Q3UGP8**	Putative Dol-P-Glc:Glc(2)Man(9)GlcNAc(2)-PP-Dol alpha-1,2-glucosyltransferase	Alg10b	2	4,2	8,712423824
**P61982**	14-3-3 Protein gamma; 14-3-3 protein gamma, N-terminally processed	Ywhag	2	14,2	8,627606838
**Q9CYN2**	Signal peptidase complex subunit 2	Spcs2	2	13,3	8,601622241
**Q91WQ5**	TAF5-like RNA polymerase II p300/CBP-associated factor-associated factor 65 kDa subunit 5L	Taf5l	2	3,7	8,587815013
**Q9ER73**	Elongator complex protein 4	Elp4	2	6,6	8,514871825
**Q6PDG5**	SWI/SNF complex subunit SMARCC2	Smarcc2	2	3,7	8,502036164
**P16546**	Spectrin alpha chain, non-erythrocytic 1	Sptan1	2	1,3	8,492574264
**Q8K2V6**	Importin-11	Ipo11	2	2,4	8,485306061
**Q8CCP0**	Nuclear export mediator factor Nemf	Nemf	2	1,9	8,444932049
**Q6P5B0**	RRP12-like protein	Rrp12	2	1,9	8,426306693
**Q9Z1M8**	Protein Red	Ik	2	5,2	8,418611628
**P09021**	Homeobox protein Hox-A5	Hoxa5	2	13	8,396390716
**P28352**	DNA-(apurinic or apyrimidinic site) lyase; DNA-(apurinic or apyrimidinic site) lyase, mitochondrial	Apex1	2	10,7	8,340117144
**Q921T2**	Torsin-1A-interacting protein 1	Tor1aip1	2	5,4	8,147357583
**P05201**	Aspartate aminotransferase, cytoplasmic	Got1	2	9	8,1094128
**Q9CQY6**	Ubiquinol-cytochrome-c reductase complex assembly factor 2	Uqcc2	2	18,4	8,096240766
**Q9CZX9**	ER membrane protein complex subunit 4	Emc4	2	15,8	7,912769606
**Q6ZQ11**	Chondroitin sulfate synthase 1	Chsy1	2	4,6	7,654993973
**Q9JHS4**	ATP-dependent Clp protease ATP-binding subunit clpX-like, mitochondrial	Clpx	2	4,4	7,632413641
**Q8R2K4**	TAF6-like RNA polymerase II p300/CBP-associated factor-associated factor 65 kDa subunit 6L	Taf6l	2	3,2	7,447083226
**O88738**	Baculoviral IAP repeat-containing protein 6	Birc6	2	0,7	7,362206855
**P26883**	Peptidyl-prolyl cis-trans isomerase FKBP1A	Fkbp1a	2	25	7,275938239

**Q7TMS5**	ATP-binding cassette sub-family G member 2	Abcg2	2	3,3	7,231605208
**Q9CQ92**	Mitochondrial fission 1 protein	Fis1	2	15,8	7,111865964
**J3QN89**		Aamp	2	6	7,018144529
**Q80YX1**	Tenascin	Tnc	2	0,9	6,885330315
**E9PY39; Q9D2M8; Q9CZY3**	Ubiquitin-conjugating enzyme E2 variant 2; Ubiquitin-conjugating enzyme E2 variant 1	Gm20431;Ube2v2;Ube2v1	2	4,9	6,864557606
**Q99MN1**	Lysine--tRNA ligase	Kars	2	4,4	6,28771238
**Q9Z1B3**	1-Phosphatidylinositol 4,5-bisphosphate phosphodiesterase beta-1	Plcb1	2	2,1	6,159649367
**P10711**	Transcription elongation factor A protein 1	Tcea1	2	8,3	6,066369089
**Q9ERA6**	Tuftelin-interacting protein 11	Tfip11	2	2,5	2,228093832
**Q9D8Y1**	Transmembrane protein 126A	Tmem126a	2	10,7	1,729130444
**Q91Z67**	SLIT-ROBO Rho GTPase-activating protein 2	Srgap2	2	2,1	1,640905387
**P32883; Q61411; P08556**	GTPase KRas; GTPase KRas, N-terminally processed; GTPase HRas; GTPase HRas, N-terminally processed; GTPase NRas	Kras;Hras;Nras	2	14,3	1,618021121
**Q9D958**	Signal peptidase complex subunit 1	Spcs1	2	8,1	1,589935265
**Q6NSU3**	Glycosyltransferase 8 domain-containing protein 1	Glt8d1	2	10,5	1,545176719
**Q05186**	Reticulocalbin-1	Rcn1	2	9,8	1,377897302
**D6RFQ2; Q9Z210**	Peroxisomal membrane protein 11B	Pex11b	2	5,7	1,357120292
**Q78ZA7**	Nucleosome assembly protein 1-like 4	Nap1l4	2	12,3	1,279833488
**Q8K003**	Translation machinery-associated protein 7	Tma7	2	21,9	1,275175419
**O09061**	Proteasome subunit beta type-1	Psmb1	2	14,2	1,267559071
**G5E870**	E3 ubiquitin-protein ligase TRIP12	Trip12	2	1,1	1,183619729
**Q9DCF9**	Translocon-associated protein subunit gamma	Ssr3	2	9,7	1,141664419
**Q9D2R8**	28S Ribosomal protein S33, mitochondrial	Mrps33	2	19,8	1,123106684
**Q9CPQ1**	Cytochrome c oxidase subunit 6C	Cox6c	2	26,3	1,121314918
**Q8QZR8**	Cyclin-related protein FAM58B	Fam58b	2	9,6	1,057076052
**Q9CQ60**	6-Phosphogluconolactonase	Pgls	2	11,7	1,021826876

**Q78TU8**		Fam107a	1	6,2	12,27125875
**P01872**	Ig mu chain C region	Ighm	1	2,2	11,64218711
**Q9ES97**	Reticulon-3	Rtn3	1	1,1	11,07547915
**Q91VS7**	Microsomal glutathione S-transferase 1	Mgst1	1	10,3	10,74011804
**Q5RL79**	Keratinocyte-associated protein 2	Krtcap2	1	12,5	10,63608079
**Q9Z0S9**	Prenylated Rab acceptor protein 1	Rabac1	1	7,6	10,52816105
**Q3KNM2**	E3 ubiquitin-protein ligase MARCH5	05-Mar	1	5,8	10,45296189
**P61804**	Dolichyl-diphosphooligosaccharide--protein glycosyltransferase subunit DAD1	Dad1	1	10,6	10,32305476
**P31648**	Sodium- and chloride-dependent GABA transporter 1	Slc6a1	1	3,5	10,30571978
**P70202**	Latexin	Lxn	1	8,1	10,23038076
**P62996**	Transformer-2 protein homolog beta	Tra2b	1	3,5	10,20518233
**Q9ESK9**	RB1-inducible coiled-coil protein 1	Rb1cc1	1	0,7	10,17180229
**Q9DBZ5**	Eukaryotic translation initiation factor 3 subunit K	Eif3k	1	6,4	10,0558247
**P01867**	Ig Gamma-2B chain C region	Igh-3	1	4	10,05080096
**Q9ERN0**	Secretory carrier-associated membrane protein 2	Scamp2	1	3,6	9,899477772
**Q9NWG9**	Melanoma-associated antigen H1	Mageh1	1	9,2	9,896559463
**Q3UHX2**	28-kDa Heat- and acid-stable phosphoprotein	Pdap1	1	7,2	9,800721978
**Q9R0M8**	UDP-galactose translocator	Slc35a2	1	2,8	9,738700293
**P97384**	Annexin A11	Anxa11	1	1,8	9,718001935
**Q8R404**	Protein QIL1	Qil1	1	5,9	9,679198571
**Q791V5**	Mitochondrial carrier homolog 2	Mtch2	1	3	9,667608326
**Q9CQA6**	Coiled-coil-helix-coiled-coil-helix domain-containing protein 1	Chchd1	1	9,3	9,629775524
**P61211**	ADP-ribosylation factor-like protein 1	Arl1	1	6,1	9,502632935
**P49586; Q811Q9**	Choline-phosphate cytidylyltransferase A; Choline-phosphate cytidylyltransferase B	Pcyt1a;Pcyt1b	1	3,3	9,49026927
**P70699**	Lysosomal alpha-glucosidase	Gaa	1	2,4	9,48058827
**Q8K1A5**	Transmembrane protein 41B	Tmem41b	1	6,2	9,448013031
**Q8CGI1**	Protein FAM193A	Fam193a	1	1	9,447041888
**P27612**	Phospholipase A-2-activating protein	Plaa	1	3	9,43812648
**Q9ES89**	Exostosin-like 2	Extl2	1	3,3	9,399213827

**P70202**	Latexin	Lxn	1	8,1	10,23038076
**P62996**	Transformer-2 protein homolog beta	Tra2b	1	3,5	10,20518233
**Q9ESK9**	RB1-inducible coiled-coil protein 1	Rb1cc1	1	0,7	10,17180229
**Q9DBZ5**	Eukaryotic translation initiation factor 3 subunit K	Eif3k	1	6,4	10,0558247
**P01867**	Ig Gamma-2B chain C region	Igh-3	1	4	10,05080096
**Q9ERN0**	Secretory carrier-associated membrane protein 2	Scamp2	1	3,6	9,899477772
**Q9NWG9**	Melanoma-associated antigen H1	Mageh1	1	9,2	9,896559463
**Q3UHX2**	28-kDa Heat- and acid-stable phosphoprotein	Pdap1	1	7,2	9,800721978
**Q9R0M8**	UDP-galactose translocator	Slc35a2	1	2,8	9,738700293
**P97384**	Annexin A11	Anxa11	1	1,8	9,718001935
**Q8R404**	Protein QIL1	Qil1	1	5,9	9,679198571
**Q791V5**	Mitochondrial carrier homolog 2	Mtch2	1	3	9,667608326
**Q9CQA6**	Coiled-coil-helix-coiled-coil-helix domain-containing protein 1	Chchd1	1	9,3	9,629775524
**P61211**	ADP-ribosylation factor-like protein 1	Arl1	1	6,1	9,502632935
**P49586; Q811Q9**	Choline-phosphate cytidylyltransferase A; Choline-phosphate cytidylyltransferase B	Pcyt1a;Pcyt1b	1	3,3	9,49026927
**P70699**	Lysosomal alpha-glucosidase	Gaa	1	2,4	9,48058827
**Q8K1A5**	Transmembrane protein 41B	Tmem41b	1	6,2	9,448013031
**Q8CGI1**	Protein FAM193A	Fam193a	1	1	9,447041888
**P27612**	Phospholipase A-2-activating protein	Plaa	1	3	9,43812648
**Q9ES89**	Exostosin-like 2	Extl2	1	3,3	9,399213827
**Q61387**	Cytochrome c oxidase subunit 7A-related protein, mitochondrial	Cox7a2l	1	28,8	9,398145117
**Q99JT6**	Calfacilitin	Tlcd1	1	3,6	9,366562643
**Q8CJG1**	Protein argonaute-1	Ago1	1	1,4	9,33621643
**Q9JLV2**	Short transient receptor potential channel 4-associated protein	Trpc4ap	1	4	9,332551305
**Q9CQ90**	Uncharacterized protein C9orf85 homolog		1	16,8	9,30058136
**P16332**	Methylmalonyl-CoA mutase, mitochondrial	Mut	1	2,4	9,285956995
**P47802**	Metaxin-1	Mtx1	1	5	9,277752336
**Q9DC29**	ATP-binding cassette sub-family B member 6, mitochondrial	Abcb6	1	2,6	9,249990421

**Q9Z2A7**	Diacylglycerol O-acyltransferase 1	Dgat1	1	8,4	9,230692976
**Q6URW6**	Myosin-14	Myh14	1	1,9	9,206208529
**Q91VP7**	Transmembrane protein 101	Tmem101	1	3,5	9,200015803
**Q8R1Z9**	RING finger protein 121	Rnf121	1	4	9,158761144
**P60003**	Transcription elongation factor 1 homolog	Elof1	1	21,7	9,157094252
**Q8JZS9**	39S Ribosomal protein L48, mitochondrial	Mrpl48	1	5,2	9,144556282
**Q99ME2**	WD repeat-containing protein 6	Wdr6	1	3,2	9,097874115
**Q9D0W5**	Peptidyl-prolyl cis-trans isomerase-like 1	Ppil1	1	10,2	9,095871689
**P46061**	Ran GTPase-activating protein 1	Rangap1	1	2	9,063206305
**A2BH40**	AT-rich interactive domain-containing protein 1A	Arid1a	1	0,9	9,030004945
**Q9JKN1**	Zinc transporter 7	Slc30a7	1	2,6	8,964514154
**Q9EP78**	Carbohydrate sulfotransferase 7	Chst7	1	2,7	8,961102364
**Q9JKD3**	Secretory carrier-associated membrane protein 5	Scamp5	1	3,8	8,95991502
**O70274; Q63739**	Protein tyrosine phosphatase type IVA 2;<br>Protein tyrosine phosphatase type IVA 1	Ptp4a2;Ptp4a1	1	8,4	8,959393438
**Q8CB77**	Transcription elongation factor B polypeptide 3	Tceb3	1	1,9	8,943393929
**Q2PMX6**		Dmrtc1b	1	4,5	8,934929248
**Q9JLJ5**	Elongation of very long chain fatty acids protein 1	Elovl1	1	4,3	8,922970853
**Q9CQD1**	Ras-related protein Rab-5A	Rab5a	1	15,3	8,913308315
**Q91YW3**	DnaJ homolog subfamily C member 3	Dnajc3	1	3,4	8,912021063
**Q8JZM0**	Dimethyladenosine transferase 1, mitochondrial	Tfb1m	1	4,3	8,909923077
**Q9DAJ4**	WD repeat domain-containing protein 83	Wdr83	1	5,4	8,907852072
**P55821**	Stathmin-2	Stmn2	1	5,6	8,888621497
**Q99J09**	Methylosome protein 50	Wdr77	1	5	8,886946371
**Q01279**	Epidermal growth factor receptor	Egfr	1	1,6	8,886245286
**P05977; P09542**	Myosin light chain 1/3, skeletal muscle isoform; Myosin light chain 3	Myl1;Myl3	1	8,5	8,878112237
**O08912**	Polypeptide N-acetylgalactosaminyltransferase 1;	Galnt1	1	4,7	8,871165994

	Polypeptide N-acetylgalactosaminyltransferase 1 soluble form				
**Q3V1T4**	Prolyl 3-hydroxylase 1	Lepre1	1	1,6	8,819604323
**Q8BGT7**	Survival of motor neuron-related-splicing factor 30	Smndc1	1	8,8	8,812786992
**P61924**	Coatomer subunit zeta-1	Copz1	1	13	8,809092838
**Q9QYI6**	DnaJ homolog subfamily B member 9	Dnajb9	1	5,4	8,807419327
**Q8BLK3**	Limbic system-associated membrane protein	Lsamp	1	3,2	8,806517401
**Q8BG51**	Mitochondrial Rho GTPase 1	Rhot1	1	2,4	8,787478175
**Q64310**	Surfeit locus protein 4	Surf4	1	4,8	8,781162969
**Q8CEE7**	Retinol dehydrogenase 13	Rdh13	1	5,1	8,770531757
**P28474**	Alcohol dehydrogenase class-3	Adh5	1	4,5	8,765833067
**Q8CHP5**	Partner of Y14 and mago	Wibg	1	7,9	8,7601544
**Q8VIK2**	Mpv17-like protein 2	Mpv17l2	1	6,5	8,756589679
**P59325**	Eukaryotic translation initiation factor 5	Eif5	1	2,3	8,756189348
**Q9DCI3**	MLN64 N-terminal domain homolog	Stard3nl	1	4,3	8,750974902
**Q8BGQ1**	Spermatogenesis-defective protein 39 homolog	Vipas39	1	3,9	8,74819285
**Q80Y32**		Hmgxb4	1	2,9	8,737382563
**Q8BX10**	Serine/threonine-protein phosphatase PGAM5, mitochondrial	Pgam5	1	3,5	8,717573615
**Q6PGB8**	Probable global transcription activator SNF2L1	Smarca1	1	1,3	8,701445093
**P17427; P17426**	AP-2 complex subunit alpha-2; AP-2 complex subunit alpha-1	Ap2a2;Ap2a1	1	1	8,651733401
**P70671**	Interferon regulatory factor 3	Irf3	1	4,8	8,65047737
**O35623**	BET1 homolog	Bet1	1	9,3	8,650118303
**Q6ZQ29**	Serine/threonine-protein kinase TAO2	Taok2	1	2,3	8,64731451
**P08551**	Neurofilament light polypeptide	Nefl	1	5	8,627716262
**Q9CQW7**	Apoptogenic protein 1, mitochondrial	Apopt1	1	9,4	8,616953109
**P03899**	NADH-ubiquinone oxidoreductase chain 3	Mtnd3	1	13	8,610323573
**O70310**	Glycylpeptide N-tetradecanoyltransferase 1	Nmt1	1	2,6	8,6043679
**Q06831**	Transcription factor SOX-4	Sox4	1	5,9	8,600804955

**Q9D1Q4**	Dolichol-phosphate mannosyltransferase subunit 3	Dpm3	1	10,9	8,599950025
**D3YZZ5**		Tmed7	1	4,5	8,585713709
**Q6P5F7**	Protein tweety homolog 3	Ttyh3	1	2,7	8,578863256
**P84096**	Rho-related GTP-binding protein RhoG	Rhog	1	9,9	8,557119022
**Q3TBW2**	39S ribosomal protein L10, mitochondrial	Mrpl10	1	5,7	8,539507808
**Q80U78**	Pumilio homolog 1	Pum1	1	1,7	8,531420452
**Q8K0D7**	Tail-anchored protein insertion receptor WRB	Wrb	1	6,9	8,523757961
**Q8BUN5; Q9JIW5; Q62432**	Mothers against decapentaplegic homolog 3; Mothers against decapentaplegic homolog 9; Mothers against decapentaplegic homolog 2	Smad3;Smad9;Smad2	1	3,1	8,518771163
**P50247**	Adenosylhomocysteinase	Ahcy	1	2,5	8,517826836
**Q149F3; Q8R050**	Eukaryotic peptide chain release factor GTP-binding subunit ERF3B; Eukaryotic peptide chain release factor GTP-binding subunit ERF3A	Gspt2;Gspt1	1	2,8	8,516921276
**P84102; O88892**	Small EDRK-rich factor 2; Small EDRK-rich factor 1	Serf2;Serf1	1	13,6	8,503189699
**Q9CZX5**	PIN2/TERF1-interacting telomerase inhibitor 1	Pinx1	1	5,7	8,502792032
**Q8BHN0**	Protein phosphatase 1L	Ppm1l	1	3,6	8,499845887
**Q99P72**	Reticulon-4	Rtn4	1	2,7	8,49589499
**Q9D8B4**	NADH dehydrogenase [ubiquinone] 1 alpha subcomplex subunit 11	Ndufa11	1	16,3	8,483291798
**Q8BTV2**	Cleavage and polyadenylation specificity factor subunit 7	Cpsf7	1	2,5	8,48143619
**Q8BPE4**	Transmembrane protein 177	Tmem177	1	3,2	8,420802382
**Q8BKE6**	Cytochrome P450 20A1	Cyp20a1	1	3,9	8,384136172
**A2AM29**	Protein AF-9	Mllt3	1	2,6	8,38383387
**P03975**	IgE-binding protein	Iap	1	2	8,380201292
**Q9D855**	Cytochrome b-c1 complex subunit 7	Uqcrb	1	13,5	8,37716714
**A6X919**	Probable C-mannosyltransferase DPY19L1	Dpy19l1	1	1,9	8,365403843
**Q9DCR2**	AP-3 complex subunit sigma-1	Ap3s1	1	7,8	8,365141344
**P59108**	Copine-2	Cpne2	1	2,9	8,353455626
**Q64674**	Spermidine synthase	Srm	1	3,3	8,34638076
**Q9CQ89**	Protein CutA	Cuta	1	7,9	8,345760055

**P47754**	F-Actin-capping protein subunit alpha-2	Capza2	1	6,3	8,331767907
**O08848**	60 kDa SS-A/Ro ribonucleoprotein	Trove2	1	2	8,319717275
**Q61699**	Heat shock protein 105 kDa	Hsph1	1	1,5	8,317367388
**Q3UKJ7**	WD40 repeat-containing protein SMU1;<br>WD40 repeat-containing protein SMU1, N-terminally processed	Smu1	1	2,5	8,305240966
**Q9WTL7**	Acyl-protein thioesterase 2	Lypla2	1	7,8	8,302501844
**Q9D1C9**	Ribosomal RNA-processing protein 7 homolog A	Rrp7a	1	6,4	8,292505697
**O70378**	ER membrane protein complex subunit 8	Emc8	1	7,7	8,289926659
**Q80XI3**	Eukaryotic translation initiation factor 4 gamma 3	Eif4g3	1	0,8	8,288773813
**Q62311**	Transcription initiation factor TFIID subunit 6	Taf6	1	1,8	8,259790333
**Q99KU0**	Vacuole membrane protein 1	Vmp1	1	7,4	8,233188242
**P52503**	NADH dehydrogenase [ubiquinone] iron-sulfur protein 6, mitochondrial	Ndufs6	1	8,6	8,220088066
**F8VQH7**		Dbx2	1	4	8,209014642
**Q9R1P3**	Proteasome subunit beta type-2	Psmb2	1	5,5	8,202858456
**P83870**	PHD finger-like domain-containing protein 5A	Phf5a	1	11,8	8,198886991
**Q9EQI8**	39S ribosomal protein L46, mitochondrial	Mrpl46	1	4,9	8,185420589
**Q9R0U0**	Serine/arginine-rich splicing factor 10	Srsf10	1	6,1	8,166263528
**Q9JMB0**	G kinase-anchoring protein 1	Gkap1	1	3	8,150407357
**Q8BRF7**	Sec1 family domain-containing protein 1	Scfd1	1	3	8,148425737
**Q9DC53**	Copine-8	Cpne8	1	3,3	8,146594131
**Q9JHR7**	Insulin-degrading enzyme	Ide	1	1,2	8,146594131
**Q9CR20**	Immediate early response 3-interacting protein 1	Ier3ip1	1	24,4	8,12902537
**P62814**	V-Type proton ATPase subunit B, brain isoform	Atp6v1b2	1	2,9	8,126601235
**D3YUM8; Q9R1P1**	Proteasome subunit beta type; Proteasome subunit beta type-3	Gm4950;Psmb3	1	7,8	8,12531014
**O70333**	Cysteine-rich PDZ-binding protein	Cript	1	16,8	8,121481714
**Q8BXZ1**	Protein disulfide-isomerase TMX3	Tmx3	1	2,4	8,109360559

**O35857**	Mitochondrial import inner membrane translocase subunit TIM44	Timm44	1	3,1	8,081350281
**O88456**	Calpain small subunit 1	Capns1	1	5,6	8,055716264
**A0A0A6YVU; Q9JKV1**	Proteasomal ubiquitin receptor ADRM1	Gm9774;Adrm1	1	3,9	8,051807107
**Q9ER67**		Maged2	1	2,1	8,049685216
**O35387**	HCLS1-associated protein X-1	Hax1	1	5	8,042863176
**Q8K1C0**	Protein angel homolog 2	Angel2	1	3,3	8,035238993
**Q8C156**	Condensin complex subunit 2	Ncaph	1	1,4	8,035018995
**P32921**	Tryptophan-tRNA ligase, cytoplasmic; T1-TrpRS; T2-TrpRS	Wars	1	3,3	8,024751838
**O55137**	Acyl-coenzyme A thioesterase 1	Acot1	1	4,3	7,998421189
**P97742**	Carnitine O-palmitoyltransferase 1, liver isoform	Cpt1a	1	1,8	7,995936704
**Q8CES0**	N-Alpha-acetyltransferase 30	Naa30	1	4,1	7,99004722
**Q8BFQ6**	Disrupted in renal carcinoma protein 2 homolog	Dirc2	1	3,1	7,969300173
**B2RQG2**		Phf3	1	0,6	7,95011843
**Q9D6J6**	NADH dehydrogenase [ubiquinone] flavoprotein 2, mitochondrial	Ndufv2	1	4	7,923862268
**Q8BMJ2**	Leucine--tRNA ligase, cytoplasmic	Lars	1	2,2	7,922138369
**Q9D1E8**	1-Acyl-sn-glycerol-3-phosphate acyltransferase epsilon	Agpat5	1	5,8	7,921543444
**Q9D115**	Zinc-finger protein 706	Znf706	1	14,5	7,915819639
**Q8C1D8**	Protein IWS1 homolog	Iws1	1	1,6	7,906890596
**P0C0A3**	Charged multivesicular body protein 6	Chmp6	1	6,5	7,899356923
**Q7TT37**	Elongator complex protein 1	Ikbkap	1	0,9	7,883681905
**Q99J47**	Dehydrogenase/reductase SDR family member 7B	Dhrs7b	1	2,8	7,872951851
**Q9EQM6**	Microprocessor complex subunit DGCR8	Dgcr8	1	1,6	7,868822555
**Q8C0I1**	Alkyldihydroxyacetonephosphate synthase, peroxisomal	Agps	1	3,1	7,855927425
**Q8R0F5**	RNA-binding motif protein, X-linked 2	Rbmx2	1	4	7,852997588
**Q61979**	Neuronatin	Nnat	1	12,3	7,838699702
**Q99NH2**	Partitioning defective 3 homolog	Pard3	1	1,6	7,823239844
**Q5SSI6**	U3 small nucleolar RNA-associated protein 18 homolog	Utp18	1	2,2	7,814294058

**Q61187**	Tumor susceptibility gene 101 protein	Tsg101	1	3,1	7,80928581
**Q9D8V7**	Signal peptidase complex catalytic subunit SEC11C	Sec11c	1	10,4	7,806195147
**Q9Z0J0**	Epididymal secretory protein E1	Npc2	1	10,7	7,800835204
**Q8BXJ9**	Transmembrane protein 62	Tmem62	1	3,1	7,781753122
**Q99L43**	Phosphatidate cytidylyltransferase 2	Cds2	1	3,8	7,778011402
**Q6ZPR5**	Sphingomyelin phosphodiesterase 4	Smpd4	1	1,8	7,741871424
**Q9D8U2**	Transmembrane protein 41A	Tmem41a	1	4,5	7,717882018
**Q3UQ44**	Ras GTPase-activating-like protein IQGAP2	Iqgap2	1	0,6	7,711701384
**Q7TMF3**	NADH dehydrogenase [ubiquinone] 1 alpha subcomplex subunit 12	Ndufa12	1	8,3	7,699329526
**Q99K43**	Protein regulator of cytokinesis 1	Prc1	1	1,7	7,681660088
**D3Z0K6**		Rsbn1l	1	1,8	7,648824922
**Q9D0R2**	Threonine-tRNA ligase, cytoplasmic	Tars	1	1,4	7,623735202
**Q9Z2I8**	Succinyl-CoA ligase [GDP-forming] subunit beta, mitochondrial	Suclg2	1	5,8	7,617283788
**P30416**	Peptidyl-prolyl cis-trans isomerase FKBP4;<br>Peptidyl-prolyl cis-trans isomerase FKBP4, N-terminally processed	Fkbp4	1	2	7,596711567
**Q8K354**	Carbonyl reductase [NADPH] 3	Cbr3	1	4	7,576673293
**A2AAY5**	SH3 and PX domain-containing protein 2B	Sh3pxd2b	1	2,1	7,574858391
**Q9JM76**	Actin-related protein 2/3 complex subunit 3	Arpc3	1	6,2	7,564606811
**Q9EP72**	ER membrane protein complex subunit 7	Emc7	1	10,4	7,525129251
**P28571**	Sodium- and chloride-dependent glycine transporter 1	Slc6a9	1	1,4	7,489687429
**Q8VC48**	Peroxisome assembly protein 12	Pex12	1	7	7,487277313
**Q9JL26**	Formin-like protein 1	Fmnl1	1	2,7	7,484540972
**P70257**	Nuclear factor 1 X-type	Nfix	1	10	7,481718719
**Q8BZQ7**	Anaphase-promoting complex subunit 2	Anapc2	1	1,1	7,463033855
**Q505F5**	Leucine-rich repeat-containing protein 47	Lrrc47	1	3,1	7,462297766
**Q6ZQ73**	Cullin-associated NEDD8-dissociated protein 2	Cand2	1	1,9	7,444517993
**B9EJ86**	Oxysterol-binding protein	Osbpl8	1	1,2	7,433543714

**Q3U186**	Probable arginine-tRNA ligase, mitochondrial	Rars2	1	2,1	7,406077422
**Q9JKK7**	Tropomodulin-2	Tmod2	1	4	7,378771701
**Q9D8B3**	Charged multivesicular body protein 4b	Chmp4b	1	6,2	7,369902646
**P58854**	Gamma-tubulin complex component 3	Tubgcp3	1	1,5	7,34029521
**Q8K0H5**	Transcription initiation factor TFIID subunit 10	Taf10	1	11,5	7,296182058
**Q9JME5**	AP-3 complex subunit beta-2	Ap3b2	1	3,5	7,270435501
**Q01405**	Protein transport protein Sec23A	Sec23a	1	1,7	7,264911693
**Q80VE5**		Tbc1d22b	1	2,6	7,24431626
**Q8R0G9**	Nuclear pore complex protein Nup133	Nup133	1	1,8	7,204179852
**Q99LS0**	Augurin	Ecrg4	1	7,4	7,188836073
**Q8BMQ2**	General transcription factor 3C polypeptide 4	Gtf3c4	1	3,5	7,184181096
**Q8BUV8**	Protein GPR107	Gpr107	1	2	7,14689956
**P80205**	Homeobox protein OTX1	Otx1	1	3,4	7,136068312
**Q99JH8**	ER lumen protein-retaining receptor 1	Kdelr1	1	9	7,096240766
**P97858**	Solute carrier family 35 member B1	Slc35b1	1	3,1	7,085339669
**Q9QYC0**	Alpha-adducin	Add1	1	2,4	7,085020924
**Q99KI3**	ER membrane protein complex subunit 3	Emc3	1	6,5	7,06371864
**Q9QXK3**	Coatomer subunit gamma-2	Copg2	1	3,3	7,062639828
**Q9JM99**	Proteoglycan 4;<br>Proteoglycan 4 C-terminal part	Prg4	1	1,6	7,001464494
**Q9Z0E0**	Neurochondrin	Ncdn	1	2,1	6,988457473
**Q02111**	Protein kinase C theta type	Prkcq	1	1,1	6,984475425
**Q3UU43**		Chpf2	1	2,5	6,970738639
**P36916**	Guanine nucleotide-binding protein-like 1	Gnl1	1	2	6,955708021
**Q80UU9**	Membrane-associated progesterone receptor component 2	Pgrmc2	1	11,5	6,947432389
**Q7M6Y3; Q61548**	Phosphatidylinositol-binding clathrin assembly protein;<br>Clathrin coat assembly protein AP180	Picalm;Snap 91	1	1,8	6,928133408
**Q99J27**	Acetyl-coenzyme A transporter 1	Slc33a1	1	2	6,895302621
**Q6PD26**	GPI transamidase component PIG-S	Pigs	1	3,8	6,87282876

**Q8BH73**	Glutaminyl-peptide cyclotransferase-like protein	Qpctl	1	3,7	6,869254527
**Q61001**	Laminin subunit alpha-5	Lama5	1	0,3	6,837312556
**Q8BH64**	EH domain-containing protein 2	Ehd2	1	4,6	6,822220272
**Q8CI94**	Glycogen phosphorylase, brain form	Pygb	1	1,4	6,761817143
**Q8BQM4**	HEAT repeat-containing protein 3	Heatr3	1	1,6	6,708325207
**P28184**	Metallothionein-3	Mt3	1	17,6	6,656782364
**P17710**	Hexokinase-1	Hk1	1	1,5	6,601221086
**P70195**	Proteasome subunit beta type-7	Psmb7	1	6,9	6,569293607
**Q924C1**	Exportin-5	Xpo5	1	0,8	6,491211756
**Q9D0F9**	Phosphoglucomutase-1	Pgm1	1	3,2	6,409577626
**Q8BIX3**	ARL14 effector protein	Arl14ep	1	5,8	6,235363277
**P58742**	Aladin	Aaas	1	2,9	6,208029464
**Q80TL7**	Protein MON2 homolog	Mon2	1	0,6	5,936567275
**Q8BS95**	Golgi pH regulator	Gpr89a	1	5,9	5,694768783
**Q8JZM7**	Parafibromin	Cdc73	1	3,8	5,658240051
**Q9CQN6**	Transmembrane protein 14C	Tmem14c	1	25,4	5,585413272
**Q9JI33**	Netrin-4	Ntn4	1	3	4,032139453
**P0DN91; P0DN90; P0DN89**			1	8,9	2,624162979
**P18828**	Syndecan-1	Sdc1	1	6,4	1,877080242
**P62748**	Hippocalcin-like protein 1	Hpcal1	1	14,5	1,869411629
**Q9CRC0**	Vitamin K epoxide reductase complex subunit 1	Vkorc1	1	8,1	1,632703995
**Q9CQC9; P36536**	GTP-binding protein SAR1b;GTP-binding protein SAR1a	Sar1b;Sar1a	1	5,6	1,514517971
**Q8BHL7**	CDC42 small effector protein 1	Cdc42se1	1	15	1,297153202
**A0A1Y7VKT9; Q9EPV8**	Ubiquitin-like protein 5	Ubl5	1	12,3	1,160664742
**Q6ZWY3**	40S Ribosomal protein S27-like	Rps27l	1	23,8	1,144222615
**P68134; P68033; P63268; P62737**	Actin, alpha skeletal muscle; Actin, alpha cardiac muscle 1; Actin, gamma-enteric smooth muscle; Actin, aortic smooth muscle	Acta1;Actc1; Actg2;Acta2	1	33,2	1,10318585
**P35762**	CD81 antigen	Cd81	1	9,7	1,0330895
**Q3UV17**	Keratin, type II cytoskeletal 2 oral	Krt76	1	12,1	1,013131994
**F6ZDS4**	Nucleoprotein TPR	Tpr	248	69,6	10,55681407
**Q62167;P16381**	ATP-dependent RNA helicase DDX3X; Putative ATP-dependent RNA helicase Pl10	Ddx3x;D1Pas1	13	60,1	1,010507342

**O35691**	Pinin	Pnn	21	28,6	2,407936207
**P07356**	Annexin A2	Anxa2	17	46,3	1,007189389
**P63276**	40S Ribosomal protein S17	Rps17	16	74,8	1,045357657
**Q9WTS2**	Alpha-(1,6)-fucosyltransferase	Fut8	11	24,2	1,165357074
**Q920E5; A0A0G2JEA5**	Farnesyl pyrophosphate synthase	Fdps	10	33,7	1,492091583
**P62301**	40S Ribosomal protein S13	Rps13	10	52,3	1,014573556
**P61514**	60S Ribosomal protein L37a	Rpl37a	7	59,8	1,615189326
**Q99K85**	Phosphoserine aminotransferase	Psat1	6	16,8	12,80598564
**Q9CR67**	Transmembrane protein 33	Tmem33	6	24,7	2,915590831
**Q91V41**	Ras-related protein Rab-14	Rab14	6	30,7	2,355994031
**Q6ZWV7**	60S ribosomal protein L35	Rpl35	6	39	1,114525816
**Q9CZB0**	Succinate dehydrogenase cytochrome b560 subunit, mitochondrial	Sdhc	5	29,6	11,64849241
**P70333**	Heterogeneous nuclear ribonucleoprotein H2	Hnrnph2	5	37,2	1,418037426
**Q8JZU2**	Tricarboxylate transport protein, mitochondrial	Slc25a1	5	17	1,004529846
**Q61881**	DNA replication licensing factor MCM7	Mcm7	4	8,1	10,12760749
**Q91V04**	Translocating chain-associated membrane protein 1	Tram1	3	10,2	10,60186362
**P51807**	Dynein light chain Tctex-type 1	Dynlt1	3	38,9	9,905808168
**Q78IK2**	Up-regulated during skeletal muscle growth protein 5	Usmg5	2	27,6	9,655423389
**P52875**	Transmembrane protein 165	Tmem165	2	8,7	8,801805344
**Q8BY71**	Histone acetyltransferase type B catalytic subunit	Hat1	2	6	8,724002168
**Q62095;Q61496**	ATP-dependent RNA helicase DDX3Y	Ddx3y	2	35,9	1,356013408
**Q9CQX2**	Cytochrome b5 type B	Cyb5b	1	23,3	10,77313921
**Q9R0P4**	Small acidic protein	Smap	1	12,7	10,16628864
**Q9R0Q9**	Mannose-P-dolichol utilization defect 1 protein	Mpdu1	1	3,6	10,11517373
**Q78XF5**	Oligosaccharyltransferase complex subunit OSTC	Ostc	1	8,1	9,336394978
**Q99JX4**	Eukaryotic translation initiation factor 3 subunit M	Eif3m	1	5,9	8,605775805
**Q7TN29**	Stromal membrane-associated protein 2	Smap2	1	3	8,33998358

**Q3TDN2**	FAS-associated factor 2	Faf2	1	4	8,298429211
**P62821**	Ras-related protein Rab-1A	Rab1A	1	13,7	6,99004722
**Q62446**	Peptidyl-prolyl cis-trans isomerase FKBP3	Fkbp3	1	6,7	1,331696045
**Q9CRA4**	Methylsterol monooxygenase 1	Msmo1	1	3,8	1,012938432
**Q91V41**	Ras-related protein Rab-14	Rab14	6	30,7	2,355994031
**Q6ZWV7**	60S Ribosomal protein L35	Rpl35	6	39	1,114525816
**Q9CZB0**	Succinate dehydrogenase cytochrome b560 subunit, mitochondrial	Sdhc	5	29,6	11,64849241
**P70333**	Heterogeneous nuclear ribonucleoprotein H2	Hnrnph2	5	37,2	1,418037426
**Q8JZU2**	Tricarboxylate transport protein, mitochondrial	Slc25a1	5	17	1,004529846
**Q61881**	DNA replication licensing factor MCM7	Mcm7	4	8,1	10,12760749
**Q91V04**	Translocating chain-associated membrane protein 1	Tram1	3	10,2	10,60186362
**P51807**	Dynein light chain Tctex-type 1	Dynlt1	3	38,9	9,905808168
**Q78IK2**	Up-regulated during skeletal muscle growth protein 5	Usmg5	2	27,6	9,655423389
**P52875**	Transmembrane protein 165	Tmem165	2	8,7	8,801805344
**Q8BY71**	Histone acetyltransferase type B catalytic subunit	Hat1	2	6	8,724002168
**Q62095;Q61496**	ATP-dependent RNA helicase DDX3Y	Ddx3y	2	35,9	1,356013408
**Q9CQX2**	Cytochrome b5 type B	Cyb5b	1	23,3	10,77313921
**Q9R0P4**	Small acidic protein	Smap	1	12,7	10,16628864
**Q9R0Q9**	Mannose-P-dolichol utilization defect 1 protein	Mpdu1	1	3,6	10,11517373
**Q78XF5**	Oligosaccharyltransferase complex subunit OSTC	Ostc	1	8,1	9,336394978
**Q99JX4**	Eukaryotic translation initiation factor 3 subunit M	Eif3m	1	5,9	8,605775805
**Q7TN29**	Stromal membrane-associated protein 2	Smap2	1	3	8,33998358
**Q3TDN2**	FAS-associated factor 2	Faf2	1	4	8,298429211
**P62821**	Ras-related protein Rab-1A	Rab1A	1	13,7	6,99004722
**Q62446**	Peptidyl-prolyl cis-trans isomerase FKBP3	Fkbp3	1	6,7	1,331696045
**Q9CRA4**	Methylsterol monooxygenase 1	Msmo1	1	3,8	1,012938432

S2.2 Supplemental Table S2: P-Tpr interacting proteins of WT adult NSPCs.

Supplemental Table S2: Table showing 119 P-Tpr interacting proteins of WT adult NSPCs with a Log2 FC > 1.0.

Protein IDs	Protein names	Gene names	Unique peptides	Sequence coverage [%]	Log2 fold change (FC) of LFQ intensity (a-TPR/IgG)
**A0A140T8N2; P01642**	Ig Kappa chain V-V region L7	Gm10881	1	10,4	4,498780686
**D3YZP9**	Coiled-coil domain-containing protein 6	Ccdc6	4	12,6	1,295773258
**O09167**	60S Ribosomal protein L21	Rpl21	3	20,6	1,324105654
**O35963**	Ras-related protein Rab-33B	Rab33b	2	7,9	2,211431665
**O55135**	Eukaryotic translation initiation factor 6	Eif6	6	33,1	1,089663906
**O70589**	Peripheral plasma membrane protein CASK	Cask	2	2,1	1,361306321
**P05213;P68368 ;A0A2R8VHF3**	Tubulin alpha-1B chain; Tubulin alpha-4A chain	Tuba1b;Tub a4a	2	80,3	1,499615767
**P10126;P62631**	Elongation factor 1-alpha 1	Eef1a1	29	67,5	1,224408258
**P11440;Q14AX 6;Q69ZA1;Q04 735;Q8K0D0; Q04899;O3549 5;P97377;Q99J 95;Q3V3A1;Q 80YP0;Q64261**	Cyclin-dependent kinase 1	Cdk1	4	16,8	1,137745124
**P13707**	Glycerol-3-phosphate dehydrogenase [NAD(+)], cytoplasmic	Gpd1	7	22,6	1,363549426
**P15919**	V(D)J Recombination-activating protein 1; Endonuclease RAG1;E3 ubiquitin-protein ligase RAG1	Rag1	2	2,6	4,353341778
**P16858; A0A1D5RLD; S4R1W1;**	Glyceraldehyde-3-phosphate dehydrogenase	Gapdh; Gm3839	20	71,8	1,11882841

**Q64467; V9GX06; V9GXA7**					
**P17225; B2RU80**	Polypyrimidine tract-binding protein 1	Ptbp1	10	40	1,010126516
**P28740; Q8C0N1**	Kinesin-like protein KIF2A	Kif2a	11	14,6	1,735166157
**P30681**	High mobility group protein B2	Hmgb2	11	49,5	1,156788685
**P41731**	CD63 antigen	Cd63	1	4,6	4,721943701
**P48432; P53783; Q811W0; P53784**	Transcription factor SOX-2; Transcription factor SOX-1; Transcription factor SOX-21; Transcription factor SOX-3	Sox2;Sox1;Sox21;Sox3	2	8,2	2,272434135
**P56135**	ATP synthase subunit f, mitochondrial	Atp5j2	2	26,1	1,413832201
**P56564; O35544**	Excitatory amino acid transporter 1	Slc1a3	5	15,7	1,062490733
**P61164**	Alpha-centractin	Actr1a	3	27,7	1,599889888
**P62305**	Small nuclear ribonucleoprotein E	Snrpe	2	25	1,351564329
**P62315**	Small nuclear ribonucleoprotein Sm D1	Snrpd1	5	54,6	1,892518322
**P62320**	Small nuclear ribonucleoprotein Sm D3	Snrpd3	3	31,7	1,153755406
**P62892**	60S Ribosomal protein L39	Rpl39	1	19,6	1,008486093
**P63028**	Translationally-controlled tumor protein	Tpt1	5	34,3	1,541641712
**P63082**	V-Type proton ATPase 16 kDa proteolipid subunit	Atp6v0c	2	31,6	1,314302637
**P63325; A0A3B2W864**	40S Ribosomal protein S10	Rps10	5	33,3	1,03745558
**P68134; P68033; P62737; P63268**	Actin, alpha skeletal muscle; Actin, alpha cardiac muscle 1; Actin, aortic smooth muscle; Actin, gamma-enteric smooth muscle	Acta1;Actc1; Acta2;Actg2	1	34,7	1,282806725
**P68369; P05214**	Tubulin alpha-1A chain; Tubulin alpha-3 chain	Tuba1a; Tuba3a	1	80,3	3,370136521
**Q01730**	Ras suppressor protein 1	Rsu1	5	24,2	2,617973124
**Q3TW96;Q91YN5**	UDP-N-acetylhexosamine pyrophosphorylase-like protein 1	Uap1l1	2	5,9	1,592914243
**Q3TWW8**	Serine/arginine-rich splicing factor 6	Srsf6	4	16,8	1,372126882
**Q3TZ89**	Protein transport protein Sec31B	Sec31b	2	1,5	1,137133582

**Q3UGR5**	Haloacid dehalogenase-like hydrolase domain-containing protein 2	Hdhd2	3	16,2	1,738474817
**Q3UX10**	Tubulin alpha chain-like 3	Tubal3	1	8,3	1,442220386
**Q5SUF2**	Luc7-like protein 3	Luc7l3	3	8,8	1,391529121
**Q5SUR0**	Phosphoribosylformylglycinamidine synthase	Pfas	8	8,8	1,499241778
**Q60749; Q9WU01**	KH domain-containing, RNA-binding, signal transduction-associated protein 1	Khdrbs1	7	25,1	1,381593087
**Q61205**	Platelet-activating factor acetylhydrolase IB subunit gamma	Pafah1b3	2	7,3	1,450606584
**Q61206**	Platelet-activating factor acetylhydrolase IB subunit beta	Pafah1b2	1	3,9	2,211123412
**Q61249; Q9QZ29**	Immunoglobulin-binding protein 1	Igbp1	3	13,2	1,116800011
**Q62186**	Translocon-associated protein subunit delta	Ssr4	4	30,8	1,899341285
**Q64152**	Transcription factor BTF3	Btf3	3	27	1,260510415
**Q6P1F6; Q8BG02;Q6ZWR4; Q925E7**	Serine/threonine-protein phosphatase 2A 55-kDa regulatory subunit B alpha isoform	Ppp2r2a	6	15,4	1,541125709
**Q810B6**	Rabankyrin-5	Ankfy1	1	1,3	1,047688017
**Q8BHC4**	Dephospho-CoA kinase domain-containing protein	Dcakd	6	27,7	1,228048373
**Q8BHI7**	Elongation of very long chain fatty acids protein 5	Elovl5	1	3	1,368859514
**Q8BHT6**	Beta-1,3-Glucosyltransferase	B3galtl	2	4,5	1,522895592
**Q8BML9**		Qars	2	3,1	1,409242314
**Q8BRN9**	Coiled-coil and C2 domain-containing protein 1B	Cc2d1b	28	48,5	8,625971144
**Q8BUR4**	Dedicator of cytokinesis protein 1	Dock1	5	3,2	1,273957919
**Q8BVU5**	ADP-ribose pyrophosphatase, mitochondrial	Nudt9	12	39,4	5,148520409
**Q8BXK8;Q8VHH5**	Arf-GAP with GTPase, ANK repeat and PH domain-containing protein 1	Agap1	10	16,7	2,401060435
**Q8C2E7**	WASH complex subunit strumpellin	Kiaa0196	2	2	1,146504113
**Q8CGP5; Q8R1M2; Q8CGP7;**	Histone H2A type 1-F; Histone H2A.J; Histone H2A type 1-K;	Hist1h2af; H2afj;	1	35,4	1,928399489

**Q8BFU2; Q8CGP4; Q8CGP6; C0HKE9; C0HKE8; C0HKE7; C0HKE6; C0HKE5; C0HKE4; C0HKE3; C0HKE2; C0HKE1**	Histone H2A type 3; Histone H2A; Histone H2A type 1-H	Hist1h2ak; Hist3h2a; Hist1h2aa; Hist1h2ah			
**Q8K019**	Bcl-2-Associated transcription factor 1	Bclaf1	9	14,9	1,506306238
**Q8R010**	Aminoacyl tRNA synthase complex-interacting multifunctional protein 2	Aimp2	3	19,4	1,742938995
**Q8VDS4**	Regulation of nuclear pre-mRNA domain-containing protein 1A	Rprd1a	1	5,8	1,247481055
**Q8VGE3; Q60893**	Olfactory receptor; Olfactory receptor 151	Olfr160; Olfr151	2	2,9	2,642190614
**Q8VH51**	RNA-binding protein 39	Rbm39	4	9,2	1,238317085
**Q91W69**	Epsin-3	Epn3	1	2,7	2,039262505
**Q91W90**	Thioredoxin domain-containing protein 5	Txndc5	6	20,1	1,146315575
**Q91X97**	Neurocalcin-delta	Ncald	3	33,7	3,177778292
**Q91Z69**	SLIT-ROBO Rho GTPase-activating protein 1	Srgap1	1	2,4	1,309674627
**Q921F4**	Heterogeneous nuclear ribonucleoprotein L-like	Hnrnpll	11	23,2	1,043987411
**Q922P9**	Putative oxidoreductase GLYR1	Glyr1	4	9,3	1,405402295
**Q925I1**	ATPase family AAA domain-containing protein 3	Atad3	4	6,3	2,243947744
**Q925N0**	Sideroflexin-5	Sfxn5	4	13,5	1,736791155
**Q99KN9**	Clathrin interactor 1	Clint1	20	28,4	3,235505151
**Q99M28**	RNA-binding protein with serine-rich domain 1	Rnps1	2	7,5	1,557158456
**Q9CQT1**	Methylthioribose-1-phosphate isomerase	Mri1	3	9,2	1,489087194
**Q9CWF2**	Tubulin beta-2B chain	Tubb2b	2	79,6	2,207587926
**Q9CX86**	Heterogeneous nuclear ribonucleoprotein A0	Hnrnpa0	6	26,2	1,138289427

**Q9CY50**	Translocon-associated protein subunit alpha	Ssr1	2	8	1,16509753
**Q9CY62**	E3 ubiquitin-protein ligase RNF181	Rnf181	1	4,8	1,706513368
**Q9D024**	Coiled-coil domain-containing protein 47	Ccdc47	8	20,3	1,295595125
**Q9D3B1**	Very-long-chain (3R)-3-hydroxyacyl-CoA dehydratase 2	Hacd2	1	4,7	1,266659235
**Q9D883; Q8BGJ9**	Splicing factor U2AF 35-kDa subunit	U2af1	6	33,5	2,766214711
**Q9DBS1**	Transmembrane protein 43	Tmem43	2	6	3,031366943
**Q9EP53**	Hamartin	Tsc1	2	2	2,360121666
**Q9JJI8**	60S ribosomal protein L38	Rpl38	5	50	1,16335522
**Q9QZM0**	Ubiquilin-2	Ubqln2	3	9,2	1,23705456
**Q9Z0U1;Q9QXY1**	Tight junction protein ZO-2	Tjp2	31	29,3	2,541364885
**Q9Z1N5**	Spliceosome RNA helicase Ddx39b	Ddx39b	4	39,7	1,11493492
**Q9Z2X1**	Heterogeneous nuclear ribonucleoprotein F; Heterogeneous nuclear ribonucleoprotein F, N-terminally processed	Hnrnpf	11	45,3	2,081889252
**F6ZDS4**	Nucleoprotein TPR	Tpr	81	38,6	3,68707732
**O35691**	Pinin	Pnn	4	6,9	2,420941111
**P07356**	Annexin A2	Anxa2	11	32,4	1,266777294
**P18529;A0A075B5Q0;A0A075B5R3;A0A075B5Q4;A0A075B5Q2;A0A075B5R1;A0A075B5Q6;A0A075B5T2;A0A075B5P9;A0A075B5T3;A0A0A6YWU3;A0A075B5S5;A0A075B5R2;J3QNN6;P18525;P18524;P18530;P18526;P18527;P18528;P01801;P01802;P01799;P**	Ig heavy chain V region 5-76; Ig heavy chain V region 5-84; Ig heavy chain V region RF; Ig heavy chain V region 7-39; Ig heavy chain V region 345; Ig heavy chain V region 914; Ig heavy chain V region 6.96; Ig heavy chain V-III region J606; Ig heavy chain V-III region W3082; Ig heavy chain V-III region ABE-47N; Ig heavy chain V region AMPC1; Ig heavy chain V-III region E109; Ig heavy chain V-III region U61; Ig heavy chain V-III region T957; Ig heavy chain V-III region A4; Ig heavy chain V-III region HPC76; Ig heavy chain Mem5	Ighv5-6; Ighv7-2; Ighv5-12; Ighv5-9; Ighv5-17; Ighv5-9-1; Ighv6-3; Ighv5-4; Ighv6-6; Ighv6-7; Ighv7-4; Ighv7-3; Ighv6-4	2	12	1,890615233

**01803;P01798;P01797;P01800;P01796;P01804;P84751**					
**P51807**	Dynein light chain Tctex-type 1	Dynlt1	2	24,8	1,649027529
**P52875**	Transmembrane protein 165	Tmem165	1	5,9	1,1803963
**P61514**	60S ribosomal protein L37a	Rpl37a	3	41,3	1,244643723
**P62301**	40S ribosomal protein S13	Rps13	8	39,1	1,782793822
**P62821**	Ras-related protein Rab-1A	Rab1A	2	40,5	1,196854452
**P63276**	40S ribosomal protein S17	Rps17	6	51,9	1,660438441
**P70333**	Heterogeneous nuclear ribonucleoprotein H2	Hnrnph2	4	31,2	1,522513943
**Q3TDN2**	FAS-associated factor 2	Faf2	4	13,9	1,230129221
**Q61881**	DNA replication licensing factor MCM7	Mcm7	9	16,4	1,156083667
**Q62095;Q61496**	ATP-dependent RNA helicase DDX3Y	Ddx3y	1	28,1	1,808867741
**Q62167;P16381**	ATP-dependent RNA helicase DDX3X;Putative ATP-dependent RNA helicase Pl10	Ddx3x;D1Pas1	5	37,5	1,030478515
**Q62446**	Peptidyl-prolyl cis-trans isomerase FKBP3	Fkbp3	5	27,7	1,46042274
**Q6ZWV7**	60S ribosomal protein L35	Rpl35	6	30,1	1,338456276
**Q78IK2**	Up-regulated during skeletal muscle growth protein 5	Usmg5	2	27,6	1,4526065
**Q78XF5**	Oligosaccharyltransferase complex subunit OSTC	Ostc	1	8,1	1,291364159
**Q7TN29**	Stromal membrane-associated protein 2	Smap2	1	1,9	1,293114934
**Q8BY71**	Histone acetyltransferase type B catalytic subunit	Hat1	2	7	1,061342768
**Q8JZU2**	Tricarboxylate transport protein, mitochondrial	Slc25a1	3	9,3	1,411726788
**Q91V04**	Translocating chain-associated membrane protein 1	Tram1	2	5,6	1,292887206
**Q91V41**	Ras-related protein Rab-14	Rab14	8	45,1	1,072374102
**Q920E5;A0A0G2JEA5**	Farnesyl pyrophosphate synthase	Fdps	10	32,6	1,288873239
**Q99JX4**	Eukaryotic translation initiation factor 3 subunit M	Eif3m	4	17,4	1,699933888
**Q99K85**	Phosphoserine aminotransferase	Psat1	18	59,2	1,405771987

**Q9CQX2**	Cytochrome b5 type B	Cyb5b	3	40,4	1,174725104
**Q9CR67**	Transmembrane protein 33	Tmem33	4	15	1,42845741
**Q9CRA4**	Methylsterol monooxygenase 1	Msmo1	3	10,9	1,05664817
**Q9CZB0**	Succinate dehydrogenase cytochrome b560 subunit, mitochondrial	Sdhc	2	12,4	1,855830867
**Q9R0P4**	Small acidic protein	Smap	2	12,7	1,241030791
**Q9R0Q9**	Mannose-P-dolichol utilization defect 1 protein	Mpdu1	3	13,8	1,245829558
**Q9WTS2**	Alpha-(1,6)-fucosyltransferase	Fut8	1	1,6	2,103859235
